# Expectations of objective threats and aversive feelings in specific fears

**DOI:** 10.1038/s41598-021-00317-3

**Published:** 2021-10-21

**Authors:** James W. B. Elsey, Merel Kindt

**Affiliations:** Department of Clinical Psychology, University of Amsterdam, Amsterdam, The Netherlands

**Keywords:** Human behaviour, Emotion

## Abstract

Mistaken beliefs about danger posed by feared stimuli are considered a key factor causing and maintaining fears. Such beliefs are intriguing because many fearful people express them, but acknowledge they are untrue in reality. While previous research indicates fearful individuals may not wholly endorse their beliefs about objective threats (e.g., the spider will bite), expectations of negative *subjective* consequences (e.g., I will feel terrible) are also likely to be important. We investigated the extent to which participants’ expectations of objective and subjective threats were sensitive to manipulations that encouraged them to consider whether their expectations were likely to happen in reality. Across five online experiments (N = 560, or 727 with more liberal inclusion criteria), such manipulations produced lower expectancy ratings for objective but not subjective threats (versus participants who gave ratings without the manipulation). Most participants reported that anticipation of negative feelings was more concerning than actual danger. Hence, numerous fear-relevant expectations about objective threat—considered central in understanding why people are irrationally afraid—respond to small cognitive manipulations. Additionally, expectations of negative subjective experiences during fear-provoking encounters appear to be more consistently endorsed, and feature prominently in fearful individuals’ concerns about what will happen in a fear-relevant situation.

## Introduction

Irrational or maladaptive beliefs are integral to contemporary cognitive behavioural theories of anxiety disorders^1^. Though sometimes considered almost cognitively impenetrable stimulus–response reactions rather than calculated responses to perceived threat^2,3^, numerous studies suggest a certain logic underpins excessive fear responses. Phobic individuals report expectations of threat regarding their feared objects or situations that exceed those of non-fearful individuals^4,5^. It stands to reason that if you believe a spider will attack, then you would be afraid and avoid it. Cognitive behavioural therapies thus seek to challenge such threat-related beliefs either through verbal interventions, or by means of behavioural experiments and exposure procedures that test threat expectations against reality, with fear reduction expected to follow updated beliefs about threat^6,7^.

Such threat-related beliefs are a particularly fascinating topic of investigation because in many cases, fearful individuals may both express that they feel them to be true and acknowledge that they know, in reality, they are not. This sort of conflict or contradiction has been noted by many clinically-oriented researchers under names such as Rational-Emotional Dissociation^8^, the distinction between ‘intellectual’ and ‘emotional’ belief^9^, or differences in ‘knowing’ and ‘feeling’^10^. Under a contemporary cognitive behavioural framework^11,12^, one could understand these divergent perspectives as multiple representations of the feared stimulus that compete for control over behaviour. Though this phenomenon is widely recognized, it has received relatively little empirical attention.

A previous series of experiments aimed to investigate multiple representations of threat held by spider fearful individuals^24^. When confronted with fear-related stimuli, fearful individuals reported markedly greater expectations about the likelihood of threatening events happening than did non-fearful individuals. However, when required to more fully endorse these reported expectations, differences between fearful and non-fearful individuals were reduced or even negated. In one experiment, participants first reported the extent to which they expected several fear-related events might happen in a fear-provoking situation. Participants were subsequently asked to predict the number of fearful people the event would really happen to in what they were led to believe was a real forthcoming experiment, with a financial prize for the most accurate predictions. In a series of further behavioural experiments, participants were instructed that they would undergo a fear-provoking exposure task and asked to provide likelihood ratings for several fear-related events that might happen (e.g., a spider jumping on them). They subsequently were required to bet money on their most-believed outcome actually occurring during the task. Across experiments, fearful individuals’ responses in these betting manipulations suggested lower endorsement of their expressed beliefs than would be anticipated if they fully endorsed their initial likelihood ratings, and their responses were very similar to those with low levels of fear.

These studies corroborate the idea that fearful individuals may hold excessive expectations of threat while also being able to generate or retrieve a more realistic perspective. Although fearful individuals may recognize that objective threats (such as a spider biting or jumping on them) are less likely than they first appear, the expectation of highly unpleasant *subjective* consequences of fear-provoking encounters may also be important. Such subjective consequences were not addressed in the previous experiments. The emotional impact of fear-related situations has been noted as a prominent concern among phobic individuals, sometimes even above objective threats^13–15^. It has even been suggested that maladaptive thoughts about emotions (e.g., demanding not to feel them, believing they are unbearable) may be a key feature in anxiety and depressive disorders, further undermining perceived emotional control in fear-provoking situations^16^. Despite knowing that a spider will not attack, the expectation that one will find the encounter exceedingly unpleasant could be sufficient for maintaining avoidance and fear. While they may be exaggerated, such expectations are also more realistic than most conceptions of objective threat, because by definition fearful individuals *are* subjectively perturbed by encounters with their feared stimuli. Moreover, in some cases fearful individuals may infer the presence of danger on the basis of their anxious feelings^17^. Hence, the subjective experience surrounding exposure to feared stimuli may contribute to maladaptive behaviour both directly (because people typically wish to avoid highly unpleasant experiences, and this may be especially the case for anxious individuals) and indirectly (because the presence of anxiety may be taken to signal the presence of danger, or further undermine one’s experience of control). Understanding the degree to which such subjective expectations are endorsed, and how important fearful individuals find them, could prove fruitful for both theory and practice.

The present studies thus extended previous work of Elsey et al.^24^ by investigating the relative responsivity of a range of objective and subjective threat-related expectations to experimental manipulations intended to make people think about how realistic they are in reality. We also sought to understand the relative importance people ascribe to subjective vs. objective threats in relation to their fears. We focused on several of the most prevalent or currently-relevant, naturally-occurring fears to ensure we could achieve reasonable sample sizes of fearful individuals in a convenience sample (spiders, snakes, and needles/injections)^18,19^. In Experiment 1, people with high and low fear of needles were required to rate how likely they thought certain needle-related events were to happen to them. They then had to predict the number of phobic people these events would really happen to in a clinical experiment, with financial rewards for those who were most accurate. Events could be split into subjective (e.g., “the experience will be extremely unpleasant”) and objective threat (e.g., “the needle will miss the target vein and damage my arm or cause me to bleed a lot”). We anticipated that, when providing initial likelihoods, fearful individuals would estimate the probability of both subjective and objective threats higher than low fear individuals. We expected these differences to be reduced or nullified when predicting what would actually happen with phobic participants undergoing the experience: fearful individuals could recognize that their initial beliefs about objective threat might be overestimations, and both groups would recognize that fearful individuals really would have negative subjective experiences. Fearful participants were also asked if they thought they might have given lower probabilities when making predictions for their bets relative to their initial responses for objective threats. We expected that among those who identified this behaviour in themselves, the majority would say it was “Because when I think about these things really happening, I know they are unlikely” (vs. “Because bad things are really more likely to happen to me than to other people”)—thus indicating a level of insight into the irrationality of their objective threat-related beliefs.

In Experiments 2 to 5, spider, needle, and snake fearful individuals were similarly presented with a range of possible outcomes that might happen to them if exposed to their feared object or situation. Again, outcomes could be separated into subjective (e.g., “I will be so scared that it just feels absolutely terrible”) and objective threats (e.g., “The spider will bite me”). Participants were randomly split to receive either the basic instructions—simply to make their estimates of the probability—or to receive a hypothetical ‘reality cheque’. In this condition, participants received the additional instruction that, before making each of their estimates, they should think: “Would I bet on this event actually happening in reality?”. We did not know whether or not this manipulation would affect the subjective outcomes, but we expected that the objective outcomes would be *more* strongly affected: participants receiving the bet manipulation were anticipated to express lower probabilities than those who did not for objective outcomes, and the magnitude of this effect would be greater for objective than subjective events.

In all Experiments, we also asked participants what they would find worse when encountering their feared stimulus—the objective threat posed, or how the situation would make them feel. We anticipated that most fearful individuals would select ‘how the process would make them feel’, as they can acknowledge that little objective threat is posed by feared stimuli, while still anticipating that an encounter will be very unpleasant for them.

## Methods

### Ethical approval

All procedures were approved by the University of Amsterdam ethical review board (2020-CP-12531). The research was performed in accordance with relevant guidelines/regulations, and participants gave informed consent.

### Recruitment and exclusion criteria

Data were collected from residents of the USA on Amazon Mechanical Turk (MTurk), participating in exchange for $3.55. MTurk is an online marketplace where ‘requesters’ (researchers) can pay participants to work on ‘Human Intelligence Tasks’ (experiments/surveys). When appropriate measures are taken to screen out low quality responses, MTurk has been shown to provide access to a large pool of participants with quality comparable or even superior to typical subject pools or community samples^20–22^. We employed several quality checks/exclusion criteria: a high approval rate on previously completed tasks on Mechanical Turk, IP screening^20^, checking for consistency between age and year of birth, consistency in reported country of residence, two multiple choice attention checks asking about features of the described task, ensuring all questions had been answered, and a comprehension check in which participants briefly described what they had been asked to do. In Experiments 3 and 4, an additional multiple-choice comprehension check was included, asking Bet participants to identify the statement that best described their additional instructions. These criteria were given scores that were multiplied together to create a tier system of inclusions: 2 = highly reliable, used in main analyses; 1 = some errors or concerns (“Sensitivity analyses” section of the supplement features analyses with these participants included to show our findings are not highly sensitive to these choices), and < 1 = insufficiently reliable for inclusion in any analyses. Table 1 shows participants in each inclusion tier across experiments. More detail on these criteria is provided in Supplementary section *Exclusion Criteria and MTurk Settings*.

**Table 1:** Inclusion tiers and sample sizes for experiments 1–5.

	Inclusion tier			Tier 2	
	< 1	1 (1 + 2)	2	High/control	Low/bet
Experiment 1—Needles	83	70 (203)	133	60	73
Experiment 2—Spiders	39	30 (140)	110	56	54
Experiment 3—Spiders	98	24 (201)	177	101	76
Experiment 4—Needles	77	32 (117)	85	46	39
Experiment 5—Snakes	87	11 (66)	55	25	30
VPN screened/quit out	123	–	–	–	–
Siphoned to other study	470	–	–	–	–

Tier < 1 = excluded from all analyses or for separate study, Tier 1 = included in sensitivity analyses, Tier 2 = most stringent inclusion for main analyses below.

Participants were asked to indicate if they believed they were more afraid of spiders, needles/injections, or snakes, than the average person. At signup, in the informed consent, and when asked these questions, participants were assured that they would be able to take part and receive full compensation even if they did not report having any of the fears listed. If they selected one of the fears, they were assigned to that fear type’s corresponding experiment (each experiment is detailed below). When running Experiments 3 and 4, we added a further option for fear of heights, and participants who selected this option or ‘none of these fears’ were siphoned off to an alternative experiment^23^. Spider fearful participants were siphoned to this alternative experiment once we reached our stopping criteria for Experiment 3 (see “Sequential design”).

Data for Experiments 1 and 2 was collected in 4 batches between September 28th and October 2nd 2020, and for Experiments 3 and 4 in 10 batches between October 26th and February 18th 2021. Participants for Experiment 5 were collected across this entire time as few participants selected the snake fearful option. Participants who indicated they were not afraid of any of these things were assigned to the Low fear condition of Experiment 1 when it was running, or to the alternative experiment when Experiments 3 and 4 were running.

#### Participants

The sample sizes for included participants (tier 2) for each experiment are shown in Table 1. Table 2 shows the demographic composition of tier 2 participants included in all experiments. The samples were majority White, non-Hispanic, and most participants had received some education beyond high school. The sample spanned quite a large age range, with the bulk of participants being young-to-middle-aged adults.

**Table 2:** Demographic characteristics of participants in Experiments 1–5.

	Experiment 1		Experiment 2		Experiment 3		Experiment 4		Experiment 5	
	n	%	n	%	n	%	n	%	n	%
Total	133	–	110	–	177	–	85	–	55	–
American Indian, Alaskan Native, Indigenous person	–	–	–	–	1	0.6%	1	1.2%	–	–
Asian American	5	3.8%	9	8.2%	15	8.5%	8	9.4%	7	12.7%
Black/African American, non-Hispanic	3	2.3%	5	4.6%	15	8.5%	1	1.2%	7	12.7%
Hispanic or Latino	9	6.8%	3	2.7%	13	7.3%	3	3.5%	3	5.5%
White, non-Hispanic	108	81.2%	92	83.6%	129	72.9%	70	82.4%	36	65.5%
Other identification	6	4.5%	1	0.9%	3	1.7%	–	–	1	1.8%
Prefer not to say	2	1.5%	–	–	1	0.6%	2	2.4%	1	1.8%
Woman	47	35.3%	57	51.8%	90	50.8%	29	34.1%	23	41.8%
Man	86	64.7%	53	48.2%	84	47.5%	56	65.9%	32	58.2%
Other identification	–	–	–	–	3	1.7%	–	–	–	–
Less than high school degree	–	–	2	1.8%	–	–	–	–	1	1.8%
High school graduate	18	13.5%	19	17.3%	32	18.1%	6	7.1%	4	7.3%
Some college	24	18.0%	24	21.8%	31	17.5%	21	24.7%	10	18.2%
Associate degree (2-year)	12	9.0%	10	9.1%	30	16.9%	7	8.2%	7	12.7%
Bachelor's degree (4-year)	63	47.4%	46	41.8%	67	37.9%	43	50.6%	26	47.3%
Master's degree	12	9.0%	8	7.3%	13	7.3%	7	8.2%	6	10.9%
Professional degree (JD, MD)	3	2.3%	1	0.9%	3	1.7%	1	1.2%	–	–
Doctoral degree	1	0.8%	–	–	1	0.6%	–	–	1	1.8%
	Mean (sd)	Range	Mean (sd)	Range	Mean (sd)	Range	Mean (sd)	Range	Mean (sd)	Range
Age	39.5 (11.4)	22–69	38 (11.3)	21–73	34.2 (9.8)	20–73	35.8 (8.7)	20–61	39.6 (11.9)	19–69

### Materials and measures

#### Demographic information

All participants provided demographic information, indicating their age, gender, ethnicity, and education (Table 2).

#### Experiment 1

##### Belief task

Participants were asked to imagine that they were required to undergo a blood draw/injection (whichever they feared more) in their upper arm, which would be administered by a trained professional. They were then required to rate the probability of six fear-related events happening, using a sliding scale from 0 (“Certainly will NOT happen”) to 100 (“Certainly WILL happen”). The content of events was based on our previous study^24^, existing fear-related questionnaires^25^, and clinical observations of beliefs that fearful individuals report. Three events reflected objective threats (e.g., “Something will go wrong and the needle will break off or otherwise get stuck in my arm”) and three reflected subjective threats (e.g., “I will be so scared that it just feels absolutely terrible”). All stimuli are available in the supplementary section *Stimuli*.

##### Injection Phobia Scale: anxiety (IPS)^26^

The IPS is an 18-item, self-report scale used to determine fear/phobia of needles/injections/blood draws. Participants report how anxious they would be (from 0: “No anxiety”, to 4 “Maximum anxiety”) in several situations related to needles (e.g., getting a jab). Higher scores (range = 0–72) indicate greater fear of injections. The scale shows excellent reliability and validity^25,26^.

##### Bet task

Participants were informed that, as part of our research, we had conducted blood draws/injections with 50 phobic patients to expose them to their fears. Patients were said to have undergone a blood draw/injection by a trained professional while their therapist waited in another room. Their task was to predict how many of the patients had experienced six events, which matched the six events in the Belief task. For example: “When receiving the blood draw/injection, *for how many needle phobic patients do you think the needle broke or otherwise got stuck in their arm?* You can choose any number from 0 to 50”. These 0–50 ratings were doubled to 0–100 probability ratings, for direct comparison with the Belief task. Participants were told that they could win €25 by being among the participants who answered closest to what actually happened (a lottery was ultimately used, because this experiment did really not take place).

##### Post-assessment

Participants were asked what they would find worse if required to undergo a blood draw/injection: “How the process would make me feel” vs. “The danger posed by the needle/injection”. High fear participants were additionally asked if they thought they might have indicated lower likelihood ratings for objective outcomes in their Bets relative to their initial Belief ratings (Option 1 = “No”, Option 2 = “Possibly/Yes: Because bad things are really more likely to happen to me than to other phobic people”, Option 3 = “Possibly/Yes: Because when I think about these things really happening, I know they are unlikely in reality”).

#### Experiments 2 to 5

##### Abbreviated Spider Phobia Questionnaire (SPQ/SPQ-15)^27^

The SPQ-15 is a 15-item self-report questionnaire used to assess fear/phobia of spiders. Participants respond to several spider-related questions (e.g., “I shudder when I think of spiders”) in a Yes/No format. Higher scores (0–15) indicate greater fear of spiders. The scale shows high reliability and validity^27^.

##### Injection Phobia Scale: anxiety (IPS)^26^

As above.

##### Abbreviated Snake Phobia Questionnaire (SNAQ/SNAQ-12)^28^

The SNAQ-12 is a 12-item self-report questionnaire for assessing fear/phobia of snakes. Participants answer several snake-related questions (e.g., “I feel sick when I see a snake.”) in a Yes/No format. Higher scores (0–12) indicate greater fear of snakes. The scale shows good internal consistency and validity^28^.

##### Belief task

Participants were asked to imagine they were taking part in an exposure task for their respective fear. For fear of spiders, the task involved a 6–7 cm house spider positioned in the center of an enclosure. Participants were to imagine what would happen if they had to step into the enclosure alone, barefooted. They would then approach within 30 cm of the spider and touch it with a brush. For needles/injections, the task was to receive a blood draw/injection (whichever they feared most) in their upper arm. For snakes, the task was to enter an enclosure and stand 18 inches (45 cm) from the head of a 5-foot (1.5 m) boa constrictor, which they were told is non-venomous and used in demonstrations at a local zoo. They were allowed to move if the snake moved, but would be required to stand within 18 inches of the snake for one minute.

Participants had to provide probability ratings for six events that might happen. These events were derived from our previous experiments^24^, existing spider-, snake-, and needle-related questionnaires^25,28–30^, and clinical experience. Three covered objective threats/concerns (e.g., “The spider will bite me”), and three covered subjective threats/concerns (e.g., “I will be so scared that it just feels absolutely terrible”).

##### Bet vs. control manipulation

In addition to receiving the general task instructions, but before seeing the events they were required to rate, participants were randomized to either a Bet or Control condition. Participants in the Bet condition received an additional instruction when presented with the task description, asking that before giving probability ratings for each outcome, they should think about whether they would bet on the event really happening or not: “We would like you to think about your answers in a particular way: Imagine that for each event that might happen, you had to place a bet on what would actually happen in reality in the situation. You could then win money by correctly predicting what will really happen. Before answering what you think will happen, please think about whether you would bet money on the event actually happening in reality, then give your answer”.

##### Post-assessment

Participants were asked to think about what they would find worse when being exposed to a non-venomous spider (“The way the spider/snake/process makes me feel” vs. “The danger posed by the spider/snake/needle”). Participants were also asked if they believed they could do such a task (from 0 = “I definitely could NOT do it”, to 100 = “I definitely COULD do it”), and how afraid they would be performing it (from 0 = “No fear at all”, to 100 = “Worst fear imaginable”). These last two items were not included in our pre-registered analysis plan, but are included in the available data set for possible exploratory analyses.

### Procedure

Before seeing any questions, all participants were presented with an information brochure and gave informed consent to take part. Participants were then presented with the different fear options.

#### Experiment 1

If participants indicated that they thought they were more afraid than the average person of needles/injections then they were assigned to the High fear group of Experiment 1. If no fears were selected, the participant was assigned to the Low fear group of Experiment 1. Participants then performed the Belief Task, completed the IPQ, performed the Bet Task, and then answered post-assessment and demographic questions.

#### Experiments 2 to 5

If participants said they were more afraid of spiders, snakes, or needles than the average person, then they filled in the SPQ-15, SNAQ-12, or IPS respectively (recruitment for Experiment 4 Needle fearful participants took place after Experiment 1). Participants were pseudo-randomly assigned to be Control or Bet participants (using Qualtrics to aim for similar numbers of participants per group). Participants then performed the Belief Task, with the Bet group shown the bet manipulation along with the task description. Participants then answered the post-assessment and demographic questions.

### Analytic approach

#### Pre-registration

The analytic approach below was devised as an informative way to understand the data during analyses of Experiments 1 and 2, and then pre-registered—along with the custom weakly informative priors—for Experiments 3 and 4 on *AsPredicted.org,* as 'mTurk hypothetical betting study October 2020' (#50560: https://aspredicted.org/blind.php?x=zk3pb4). Experiment 5 began at the same time as Experiments 1 and 2 but, due to low participant numbers, was not analyzed until the end of all data collection for Experiments 3 and 4, using the same registered approach.

#### Analyses

Data were analysed with Bayesian regression models in *R/RStudio v1.2.5033*^31^, with the *R* package *brms v2.12.0*^32^*.* Beta regression was used, as outcome data reflected probabilities bounded at the top and bottom of the response scale. Probability ratings were converted from 0–100 to 0–1. To incorporate responses of 0 and 1, the transformation of Smithson and Verkuilen^33^ was used, which merely ‘squishes’ these responses slightly to fit within the bounds (e.g., 1 might become 0.996):

$$\left( {{\text{response}} \times \left( {N - 1} \right)\, + \,0.{5}} \right)/{\text{N}}).$$

The regression formula for Experiment 1 predicted participants’ probability estimates (either their probability rating or predicted number of phobic patients converted to a probability rating) from an interaction of Fear Level (Low vs. High, between subjects), Rating Type (Belief for self vs. Bet for phobic patients, within subjects) and Event (the 6 different possible events, within subjects), with observations nested within participants. The regression formulae for Experiments 2 to 5 were identical to one another, predicting participants’ probability ratings from their z-scored fear questionnaire scores and an interaction of Group (Control vs. Bet, between subjects) and Event (the 6 different possible events, within subjects), with observations nested within participants.

These regression parameters were used to estimate the means (aka ‘*mu*’) of the beta-distributions underpinning the observed data. *brms* additionally estimates a *phi* parameter, indicating the spread of data around the mean. As is commonly recommended^34^, custom weakly informative priors were used to speed up estimation/convergence and constrain the prior plausibility of extreme values. Priors are presented in the supplementary section *Priors* and were pre-registered after analyses of Experiments 1 and 2 to be applied in analysis of subsequent data.

Bayesian estimation enables estimation not only of a posterior distribution for means or groups differences, but also for effect sizes^35^, which are increasingly recognized as important for understanding effects^36,37^. For our key results, we present inferential statistics focusing on a robust and widely applicable effect size measure known as Probability of Superiority (PSup)^38^. More basic regression parameters (*mean* and *phi* of the beta distributions) are available as CSV tables at the OSF link. In addition, all raw data can be seen in the cumulative density figures in the results section, which include median and median absolute deviation values for descriptive purposes.

For all analyses, all MCMC chains converged (Rhat = 1) and parameters had good bulk effective sample sizes of 4000 or more. All code for PSup simulations, as well as files for every regression, are available at the OSF link.

#### Probability of superiority

Probability of superiority (PSup) is an effect size measure designed to be both easily interpretable and generalizable to a range of situations in which other common effect size metrics are not easily applied^38–40^. The PSup reflects the probability that values drawn from one distribution will be higher than (‘superior to’) values drawn from a comparison distribution. Hence, PSup values can range from 0 (none of the responses are greater), through 0.5 (observations are equally likely to be greater or smaller), up to 1 (all observations are greater). For example, a PSup of 0.67 corresponds to a 67% chance (roughly a 2:1 ratio) that a value drawn from one distribution will be greater than a value from the comparison distribution. This can be assessed both between and within subjects (in which case, within subjects observations are coupled, and the PSup reflects the proportion of participants in whom responses in one condition are superior to the other). This measure of effect size was chosen as the key metric of interest in our study because the data is represented by a beta distribution, meaning that standard parametric effect size estimates are not applicable. PSup allows us to incorporate both the mean of the beta distribution, and the spread around that mean, in our assessments of group differences, without relying on assumptions such as normality.

For the analyses below, we include both ‘raw’ PSup estimates, involving a direct estimate from the data and bootstrapped confidence intervals for each item and experimental condition, as well as Bayesian estimates of PSup derived from posterior distributions of regression model predictions using the *add_predicted_draws()* function from *tidybayes*^41^. These Bayesian estimates provide additional insight in two ways: (1) for experiments 2–5, we can incorporate fear questionnaire scores into the estimates and therefore generate PSup posterior distributions for Bet vs. Control groups when SPQ, IPS, or SNAQ scores are exactly the same across groups, thereby controlling for this source of variance, while still including noise from the general spread of the data around the mean and random variation of participant intercepts, and (2) we can directly compare different PSup posterior distributions, enabling us to assess interaction effects alongside basic effects, as explained below.

In Experiment 1, several PSup calculations are made to indicate basic effects. For example, among High and Low fear participants separately, we calculate the PSup for Belief ratings being greater than Bet ratings. For Belief ratings and Bet ratings separately, we also calculate the PSup for High fear participants providing higher ratings than Low fear participants. We can generate an interaction estimate to determine whether differences between High and Low fear participants’ ratings are greater for Belief ratings than for Bet ratings simply by subtracting the posterior distribution of the Belief PSup from that of the Bet PSup. The resulting posterior distribution is a comparison of PSup effect sizes for Beliefs vs. Bets. *Positive values indicate greater differences between High and Low fear participants in their Belief than their Bet ratings.*

In Experiments 2 to 5, PSup estimates are used to assess whether the ratings of Control participants were higher than those of Bet participants for each event. We could then determine whether the average effect of the Bet manipulation for Objective events was greater than the average effect of the Bet manipulation for Subjective events. *Positive values indicate a greater impact of the Bet manipulation on Objective than Subjective event ratings.*

Supplementary section *Probability of Superiority* also includes some visual aids to help understand PSup effect sizes.

#### Concern over danger vs. feelings

Participants’ responses to what they would find worse in a fear-relevant situation—the danger posed or their own feelings—are presented as raw percentages for each experiment below.

#### Sensitivity assessment

All analyses were re-run including participants in inclusion tier 1 to determine whether results were sensitive to reasonable variation in who was included. Tier 1 results—presented in Supplementary section *Sensitivity Analyses*—were congruent with tier 2 results presented below. In Experiment 1, a handful of IPS scores overlapped between High vs. Low fear groups. The supplement also includes a re-analysis of Experiment 1, showing comparable results to those presented below, with High and Low fear groups selected with mutually exclusive IPS scores.

#### Sequential design

After conducting Experiments 1 and 2, we aimed to replicate and extend the Experiment 2 findings. Based on results from Experiment 2 with just over 50 participants per condition, we pre-registered a sequential analytic design in which the number of participants per condition would be checked after 5 data collection batches. If there were 50 tier 2 participants per condition, the data would be analyzed and data collection would stop if the 95% HDI for the Subjective vs. Objective PSup posterior distribution excluded 0. When assessed after 5 batches, Experiment 3 had the intended sample size but the 95% HDI just touched 0 (interim analysis presented in the Supplementary section *Interim Analysis*). Data collection for Experiment 3 therefore continued for 2 further batches and resulted in the final analyses below. Data collection for Experiments 4 and 5 continued until our financial maximum of 10 batches and concluded with the final analyses below.

## Results

### Experiment 1: Needle-related beliefs among high and low fear participants

The High fear group (mean = 28.70, SD = 9.27) had reliably higher IPS scores than the Low fear group (mean = 10.07, SD = 7.72), with a Bayesian estimate of *Cohen’s d* of 2.18 (95% HDI 1.74–2.63).

**Figure 1:**
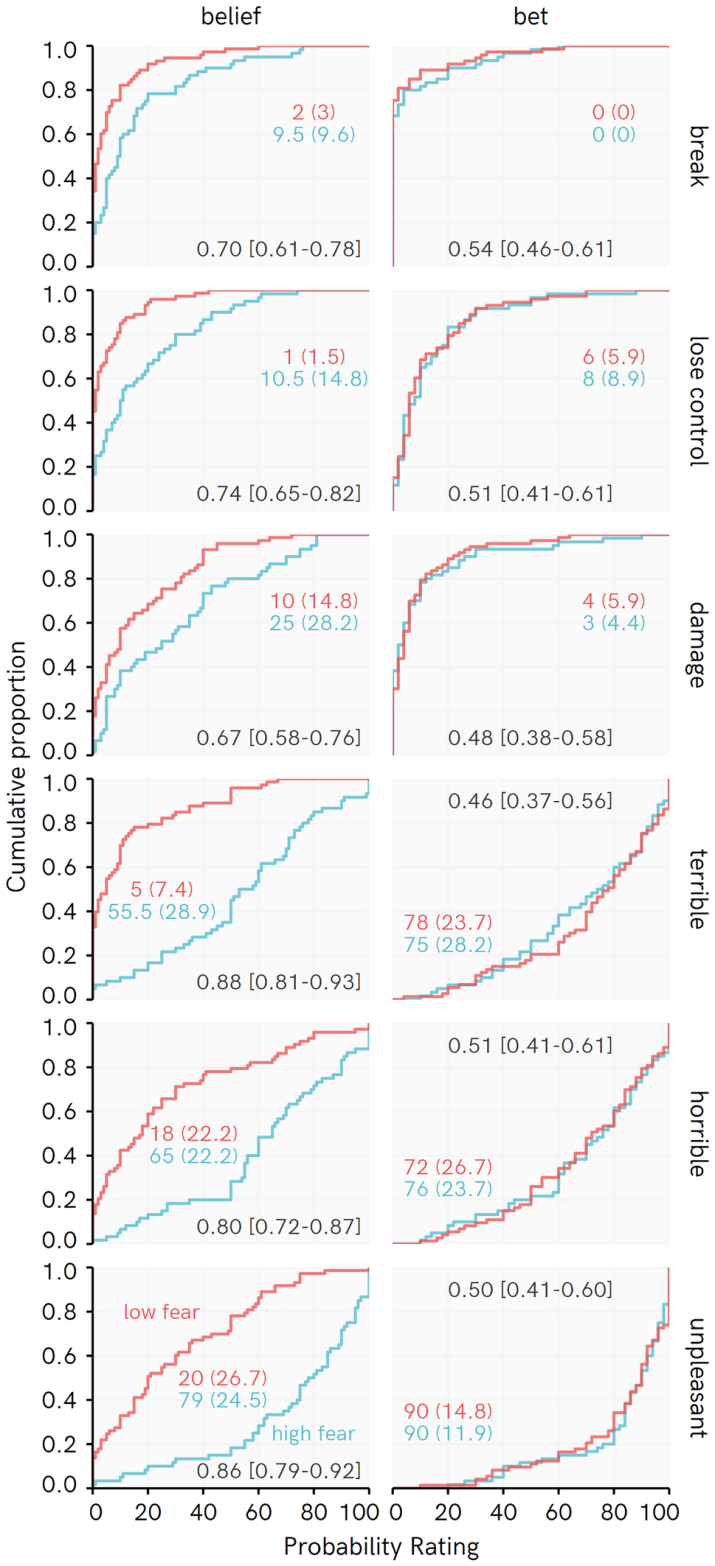
Cumulative distribution for Experiment 1, with raw probability of superiority value and bootstrapped 95% bias corrected accelerated confidence intervals. Colored numbers reflect Median and (Median absolute deviation).

As can be seen from Fig. 1, the raw data shows clear differences between High and Low fear participants in their Belief task responses across all events. Subjective events (“terrible, “horrible”, “unpleasant”) tended to show larger group differences than the objective events (“break”, “lose control”, “damage”), as visible from the larger gap between the cumulative distributions and raw PSup values. In contrast, High and Low fear participants’ responses when predicting what might actually happen (‘Bets’) closely paralleled one another: cumulative distributions overlap and the raw PSup estimates evenly span over 0.5.

**Figure 2:**
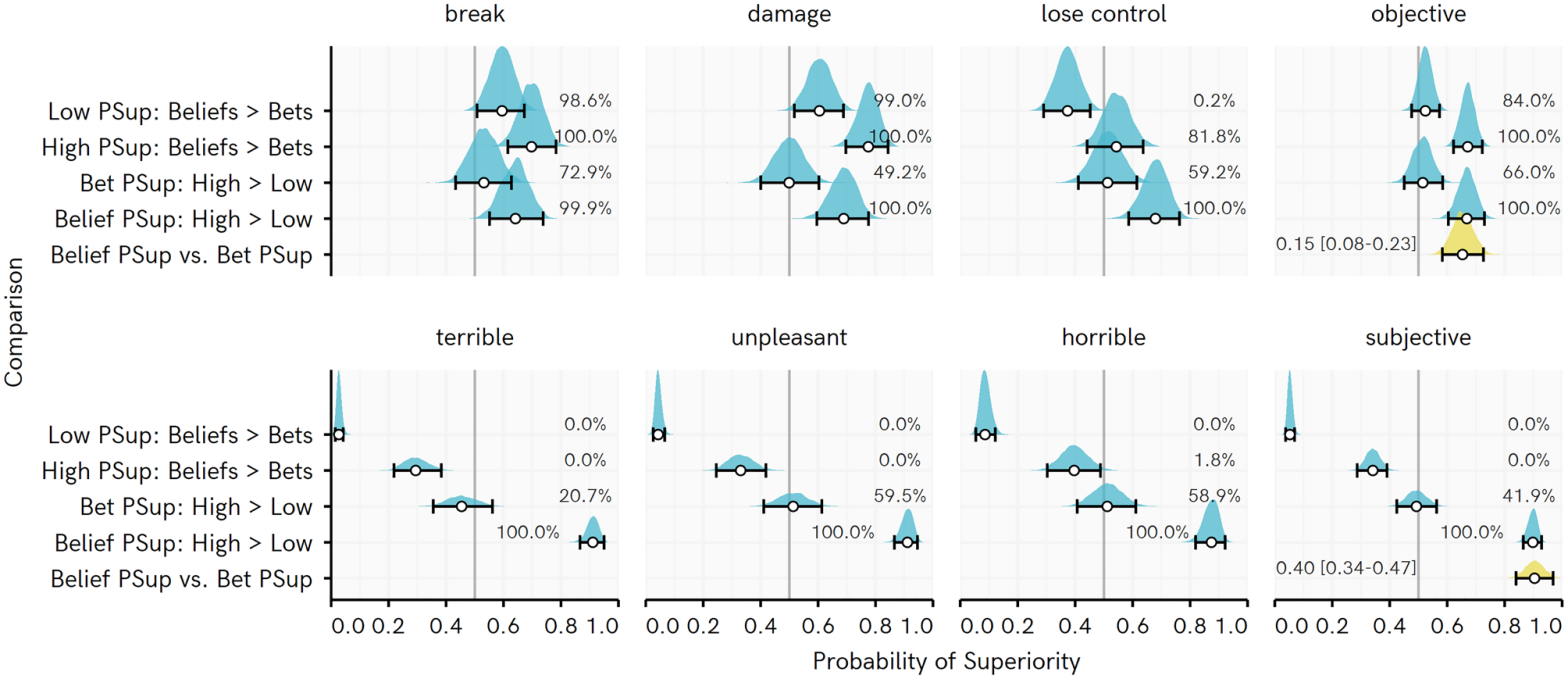
Posterior distribution of regression-based probability of superiority (PSup) estimates for Experiment 1. Percentages indicate the proportion of the posterior PSup estimate > 0.5 (e.g., for Beliefs: High—Low, 100% of the posterior for every belief is over 0.5, indicating consistently higher belief rating among high than low fear participants). Points represent posterior median, whiskers represent 95% HDI, ridge height reflects density of estimate. Belief PSup vs. Bet PSup = difference in PSup estimates for Beliefs: High > Low vs. Bets: High > Low, with values above 0 indicating a greater difference between High and Low fear participants in Beliefs than Bets, with posterior median and [95% highest density interval]. Note that position of this density ridge on the X axis has been shifted by 0.5 so that the line at 0.5 represents 0 for this posterior distribution.

This impression from the raw data is matched by Bayesian posterior estimates of PSup comparing High and Low fear groups (Fig. 2). For Belief ratings, all the posterior distributions for PSup estimates are completely or near-completely above 0.5, indicating higher estimates for the High than the Low fear group. For bets, every posterior includes substantial probability below a PSup of 0.5 (i.e., no difference, or the opposite effect, between groups). The ‘Belief PSup vs. Bet PSup’ posterior distribution serves as an assessment of a rating type × fear level interaction. This interaction is supported for both objective and subjective events, indicating that High fear participants differ more from Low fear participants in their Belief than their Bet ratings.

Within-group PSup estimates for Belief vs. Bet ratings give some insight into how respondents’ estimates changed according to how they were elicited (Beliefs > Bets in Fig. 2). Participants tended to give lower ratings for objective events occurring in the Bet relative to the Belief task. They gave higher estimates for subjective events being experienced when betting vs. their belief ratings, suggesting they recognize that phobic people would be subjectively disturbed by encountering their feared situation, even more so than themselves.

The ‘lose control’ belief provides an interesting deviation from the other two objective events, in that the PSup posterior for High fear participants did not completely exclude no differences in Belief vs. Bet ratings, and the estimate for low fear individuals indicated a greater expectation that this event would occur when betting on what would happen to phobic people. The event includes an objective outcome that explicitly includes reference to the behaviour of the fearful individual under duress. As will be elaborated on in the discussion with reference to fear of heights, these sorts of expectations might be of particular interest in understanding what people are afraid of.

Consistent with our expectations, the findings show that High fear individuals estimated threats more highly than Low fear individuals in their initially-elicited Belief ratings. Probability estimates for objective events were reduced when High fear participants were required to predict them really occurring to phobic individuals, at which point fearful participants’ estimates were not reliably different from those of Low fear participants. When betting, participants tended to agree that subjectively negative experiences would occur to phobic people.

When asked what they would find worse about a needle situation—the threat posed by the needle or how the process would make them feel—54/60 (90%) of the High fear group selected ‘how the process would make them feel’. In the Low fear group, 47/63 (64%) selected this option. Of the High fear participants who reported they thought they probably had lower estimates for objective Bet than Belief ratings (32 of 60), 29 (91%) said it was because they knew these events were unlikely in reality. However, there were 26 participants who showed a reduction for at least one objective event who did not acknowledge they had done so.

### Experiments 2 and 3: Spider-related beliefs among highly fearful participants

SPQ scores in Experiments 2 and 3 were comparable to highly fearful and phobic participants described in previous research^27^. Bet vs. Control comparisons of SPQ scores suggested similar SPQ scores between groups, with a slight tendency towards higher SPQ scores in Bet (mean 9.07, SD 3.01) than Control participants (mean 8.62, SD 2.50) in Experiment 2 (Bayesian *Cohen’s d* = 0.16, 95% HDI − 0.22 to 0.52), and a tendency towards lower SPQ scores in Bet (mean 8.51, SD 3.26) than Control participants (mean 9.21, SD 3.04) in Experiment 3 (Bayesian *Cohen’s d* = − 0.22, 95% HDI − 0.51 to 0.09). Standardized SPQ scores were included in analyses below to control for any slight group differences.

**Figure 3:**
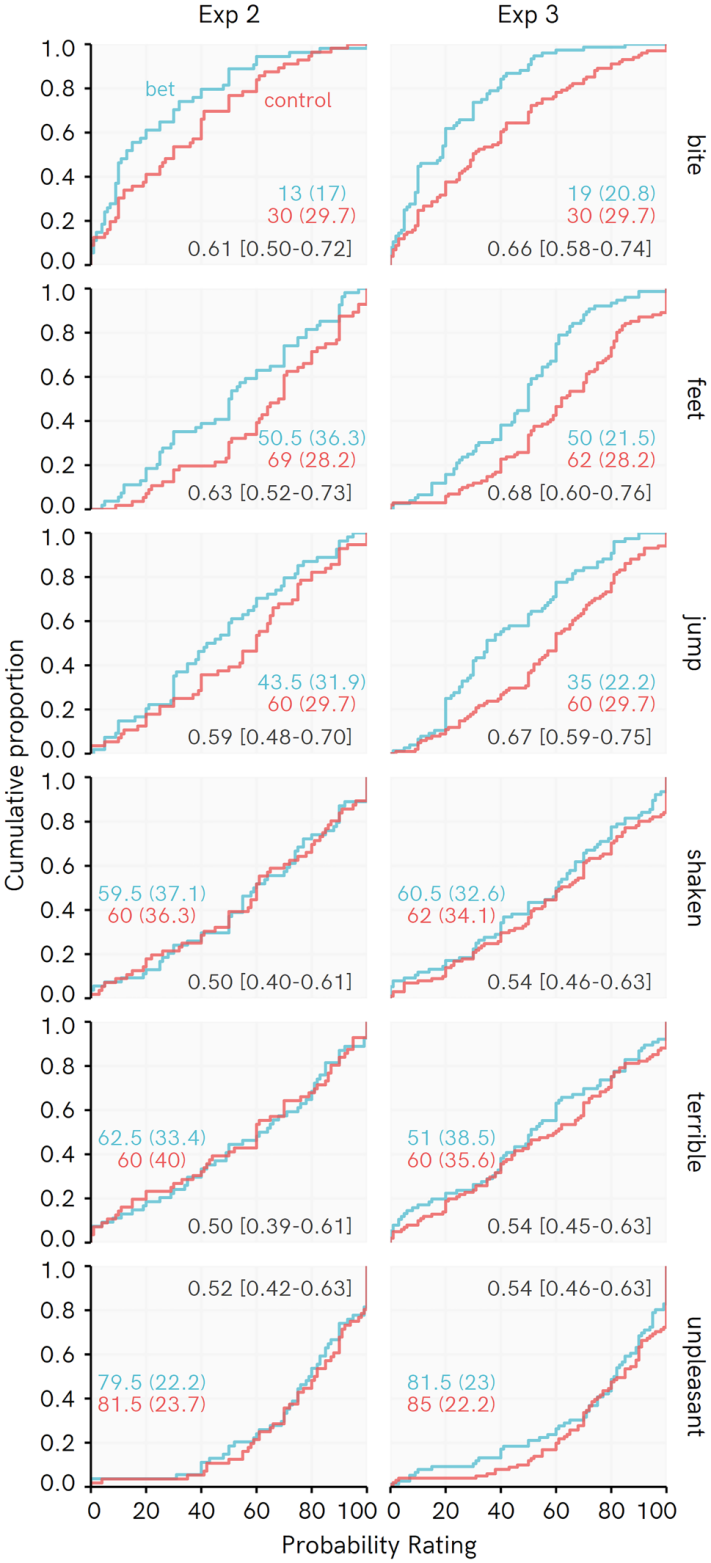
Cumulative distributions for beliefs in Experiments 2 and 3, with raw probability of superiority values and bootstrapped 95% bias corrected accelerated confidence intervals. Colored numbers reflect Median and (Median absolute deviation).

Figure 3 displays cumulative distributions of the raw data for each event in Experiments 2 and 3, and raw PSup values. Recall that a PSup value above 0.5 indicates that ratings of Control participants are greater than ratings of Bet participants. It can be seen that whereas Bet and Control participants’ responses quite closely tracked one another for subjective events (“shaken”, “terrible”, “unpleasant”), the Control participants tended to select higher probability ratings for objective events (“bite”, “feet”, “jump”).

**Figure 4:**
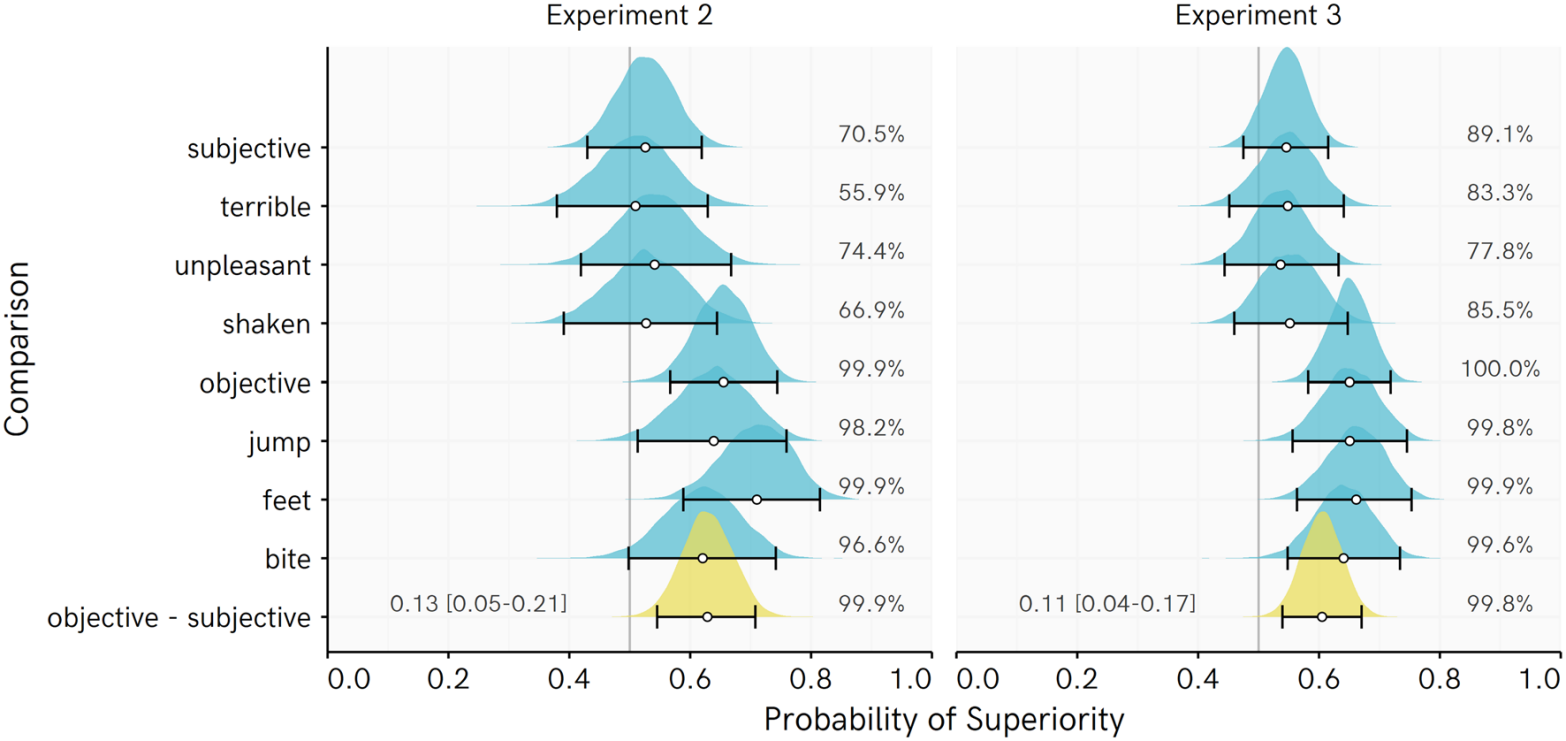
Posterior distribution of regression-based probability of superiority (PSup) estimates for Experiments 2 and 3. Each PSup value represents an estimate of the probability that a randomly picked Control score is higher than a randomly picked Bet score. Percentages indicate the proportion of the posterior PSup estimate > 0.5 (e.g., for objective events, 97% or more of every posterior is over 0.5, indicating very consistently higher ratings among Control than Bet participants). Points represent posterior median, whiskers represent 95% HDI, ridge height reflects density of estimate. Objective–Subjective = difference in PSup estimates for average of Objective beliefs vs. the average of Subjective beliefs, with values above 0 indicating a greater difference between Control and Bet participants in Objective than Subjective beliefs, with posterior median and [95% highest density interval]. Note that position of this density ridge on the X axis has been shifted by 0.5 so that the line at 0.5 represents 0 (no difference in PSup estimates) for this posterior distribution.

This interpretation of the raw data is corroborated by Bayesian posterior PSup estimates for Control participants’ probability ratings being higher for each event than those of Bet participants (Fig. 4). Across experiments, PSup posterior distributions for Objective events completely or almost completely favor higher ratings among Control than Bet participants. The posterior distributions for the Subjective events more evenly span over equivalence between the groups or even the opposite effect. There is some tendency for Bet participants to rate subjective events as less likely vs. Controls, particularly in Experiment 3.

The ‘objective–subjective’ posterior distribution serves as an assessment of a rating type × event type interaction. This distribution is reliably greater than 0 and indicates that the inclination of Control participants to rate events as more likely than Bet participants is stronger for the Objective than the Subjective events. This interaction supports the idea that fearful individuals might be more inclined to doubt the validity of their objective than their subjective threat expectations. The median estimate for this interaction is about 0.12 across the two experiments. If the PSup for subjective beliefs is about 0.55 and slightly favors higher ratings among Control participants (which is roughly the posterior estimate), the PSup for objective events would be around 0.67. Hence, we are about twice as likely to get a higher rating from a Control participant than a Bet participant for objective events. However, estimates as small as 0.04 or as large as 0.21 still receive some credibility.

When asked what they would find worse when encountering a non-venomous spider, 104/110 (95%) and 158/177 (89%) of participants selected ‘How the process would make them feel’ over ‘The danger posed by the situation’ in Experiments 2 and 3, respectively.

### Experiments 4 and 5: Needle- and snake-related beliefs among highly fearful participants

Despite continuing to our financial maximum of data collection, we did not reach the intended sample size of 50 Tier 2 participants per group for Snake or Needle fearful participants. Nevertheless, data from these experiments can be analyzed. Scores on the respective fear questionnaires were comparable to the low end of clinically phobic populations described previously^25,28^. Bet vs. Control comparisons of IPS scores suggested similar scores between groups (Bet mean 30.49, SD 11.06; Control mean 29.37, SD 9.22; Bayesian *Cohen’s d* = 0.11 [−0.33 to 0.53]). Bet vs. Control comparisons of SNAQ scores suggested similar scores between groups, with a slight tendency for lower scores among bet than control participants (Bet mean 6.40, SD 2.88; Control mean 7.16, SD 2.88; Bayesian *Cohen’s d* = − 0.26 [− 0.78 to 0.27]). Standardized fear questionnaire scores were included in analyses below to account for any slight group differences.

**Figure 5:**
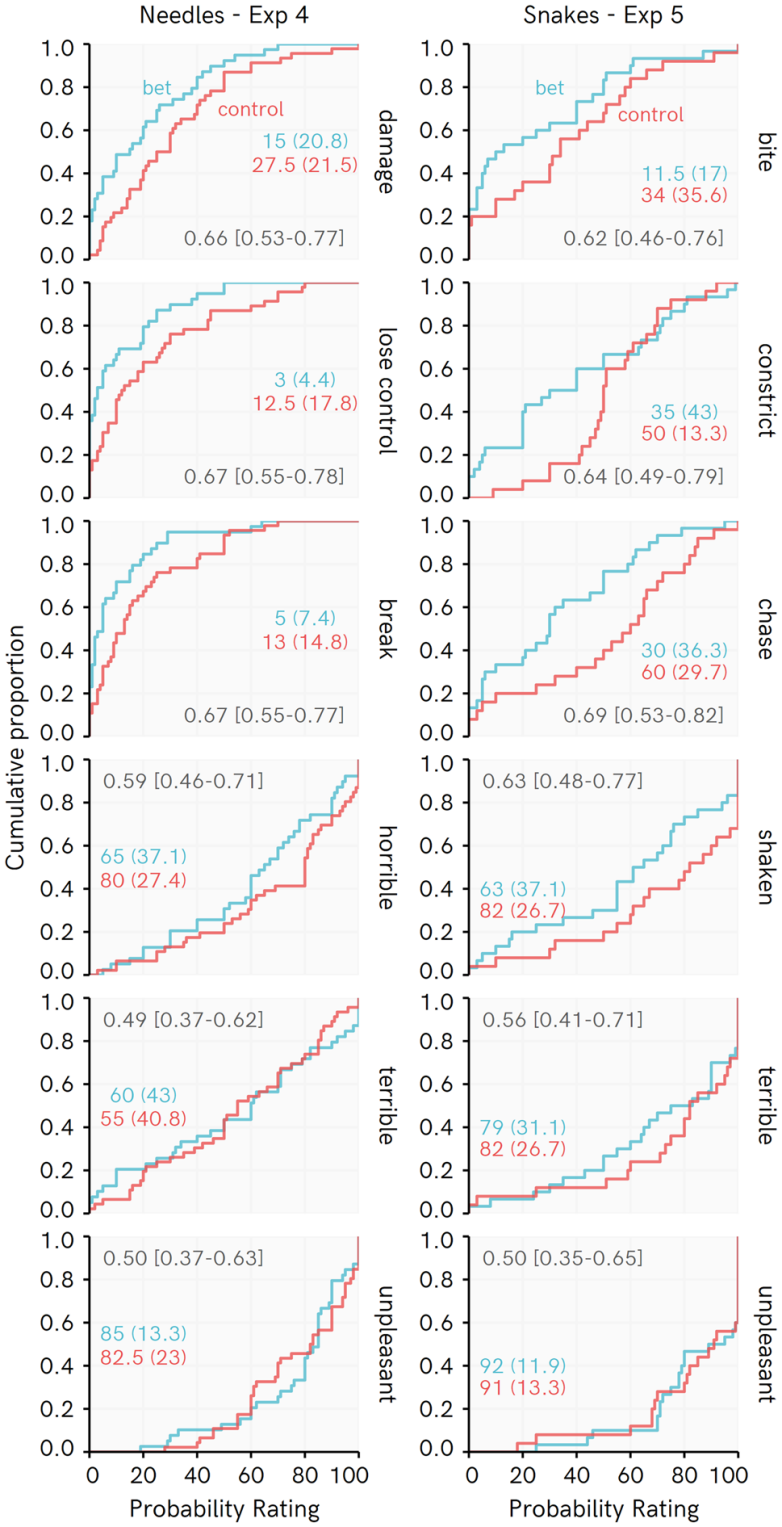
Cumulative distributions for beliefs in Experiments 4 and 5, with raw probability of superiority values and bootstrapped 95% bias corrected accelerated confidence intervals. Colored numbers reflect Median and (Median absolute deviation).

Cumulative distributions depicting responses amongst Needle and Snake fearful participants (Fig. 5), and the corresponding raw PSup measures, again suggest that differences between Bet and Control participants tended to be greater for Objective than Subjective events. This pattern is not so clean as with the larger samples in Experiments 1–3, but is supported by posterior distributions of the PSup estimates.

**Figure 6:**
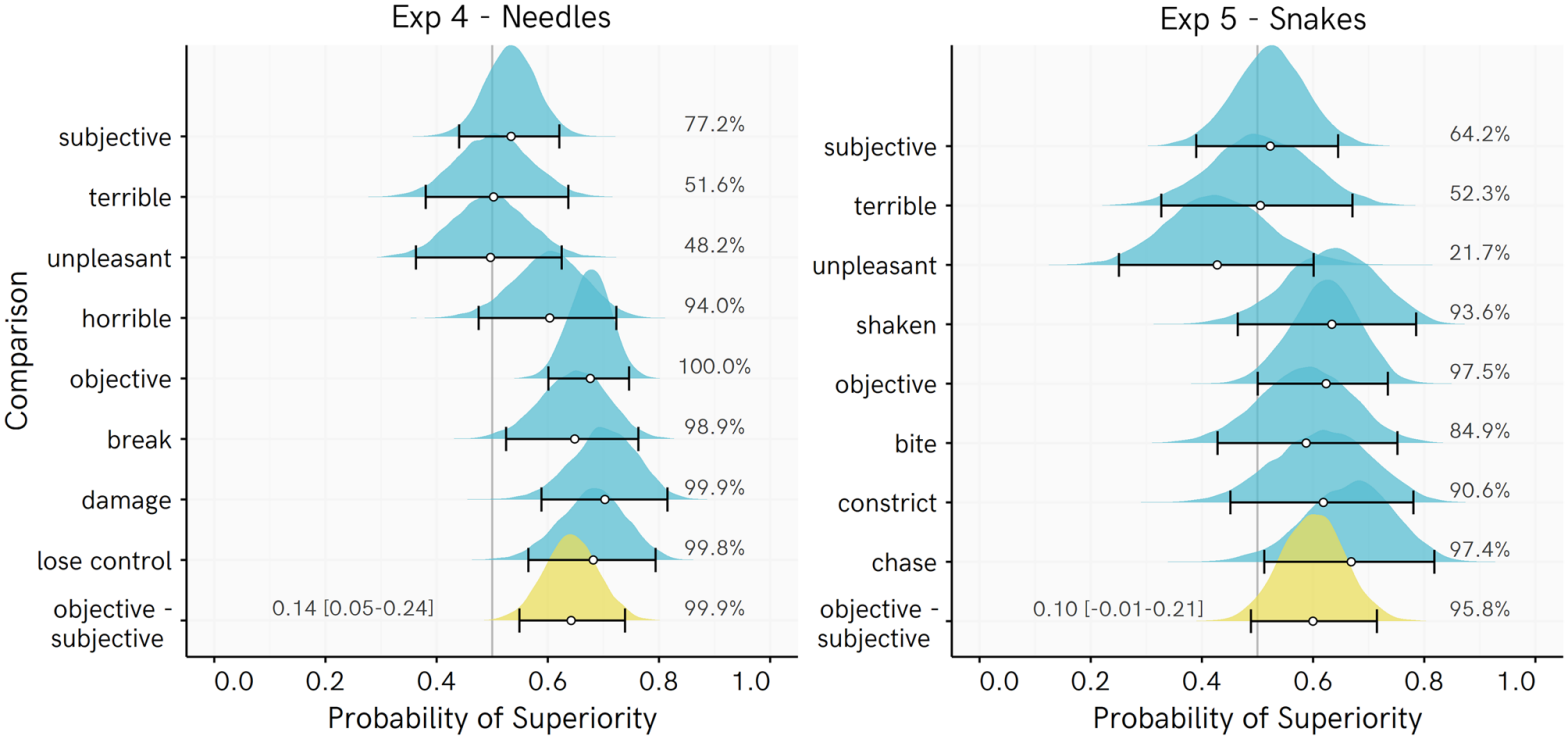
Posterior distribution of regression-based probability of superiority (PSup) estimates for Experiments 4 and 5. Each PSup value represents an estimate of the probability that a randomly picked Control score is higher than a randomly picked Bet score. Percentages indicate the proportion of the posterior PSup estimate > 0.5 (e.g., for objective events in Exp 4, 99% or more of every posterior is over 0.5, indicating very consistently higher ratings among Control than Bet participants). Points represent posterior median, whiskers represent 95% HDI, ridge height reflects density of estimate. Objective–Subjective = difference in PSup estimates for average of Objective beliefs vs. the average of Subjective beliefs, with values above 0 indicating a greater difference between Control and Bet participants in Objective than Subjective beliefs, with posterior median and [95% highest density interval]. Note that position of this density ridge on the X axis has been shifted by 0.5 so that the line at 0.5 represents 0 for this posterior distribution.

As shown in Fig. 6, the ‘Objective–Subjective’ parameter estimate for Experiment 4 is reliably positive. This indicates that, on average, Objective event ratings were more strongly affected than Subjective event ratings by the Bet manipulation among Needle fearful participants. For Snake fearful participants, this parameter is clearly tending in the same direction. When a more liberal inclusion threshold is chosen (Tier 1 and 2 participants), the posterior becomes very reliably positive (see Supplementary section *Sensitivity Analyses*). The results in Experiment 5 are therefore consistent with the other experiments, although with the achieved sample size we were not fully powered rule out an absence of this interaction with sufficient certainty. The size of these estimated effects closely parallels those of Experiments 2 and 3, although with greater uncertainty around the estimate for the Snake experiment.

When asked what they would find worse if required to receive an injection/be exposed to a non-venomous snake—how the process would make them feel, or the danger posed by the situation—70/85 (82%) of the needle participants and 41/55 (75%) snake participants indicated that the feeling was of greater concern than the danger.

## Discussion

Our five experiments supported our hypotheses that ratings for objective events would be more responsive to the bet manipulation than those of subjective events, and that unpleasant subjective outcomes would be of greater concern than objective danger for fearful individuals. In Experiment 1, compared to low fear individuals, needle fearful participants reported markedly greater expectations of negative events occurring in a needle-related situation. However, the ratings of these two groups largely converged when incentivised to predict what would actually happen to phobic people in the same situation. Fearful participants’ estimates of the likelihood of objective events typically decreased when predicting what would actually happen, whereas they expected phobic individuals really would experience the negative subjective events—even more so than themselves.

In Experiments 2 to 5 (with the caveat that the results of Experiment 5 were not so decisive due to the lower sample size), we found that asking spider, needle, or snake fearful participants to imagine having to place a bet on fear-related events occurring produced lower likelihood ratings for negative outcomes relative to a control group who simply gave their expectations. Specifically, this reduction was reliable for predictions of objective events that might occur (e.g., that the spider would jump on them), but not for predicting their subjective experiences (e.g., that they would find the exposure to be terrible). This would suggest that while fearful individuals have a tendency to recognise that the external threats they anticipate may be less likely than they at first appear, they are more convinced that unpleasant subjective experiences really will occur. This expectation is likely important, because across all experiments, the vast majority of fearful participants reported that, in a fear-provoking situation, the subjective experience was worse for them than concerns over actual danger.

The findings thus highlight both the relative fixity of fearful individuals’ expectations of negative subjective experiences and the primacy of such expectations in their concerns about fear-provoking encounters. In a cognitive framework, avoidance and fear are typically considered to be primarily the consequences of objective threat perception^1,42,43^. However, it is possible that concern over the anticipated unpleasantness of one’s subjective responses could play a role in maintaining avoidance and worry even when the validity of objective threats is doubted. Individuals with anxiety disorders have been found to be more likely than healthy controls to make catastrophic judgments about their emotions and to believe them to be unbearable^16^. Such beliefs may make anticipated negative emotions a key issue for people with specific fears. This possibility aligns with previous research and theories emphasising the importance of emotional responses in understanding fears and phobias. McNally and Steketee^13^ found that animal phobic patients mostly feared panic symptoms from a phobic encounter, with only a few fearing danger from the animal. Likewise, Lipsitz et al.^14^ found that catastrophe and harm concerns were endorsed by 25% of animal phobic patients, whereas 50% reported an emotional focus, particularly disgust^44^. However, there does appear to be variation across different objects of fear in the relative prominence of concerns about objective relative to subjective threats^14,45^. There is considerable scope for using the sort of cognitive devices we utilised here (imaginary betting, self vs. other comparisons, different forms of rating) to assess conviction in all sorts of beliefs across different fear types, and we do not assume our findings translate to all objects of fear.

Different expectations will likely also be affected differently by the cognitive manipulations we employed. Although we have separated events into subjective and objective, we are not claiming that all beliefs about subjective events will be wholeheartedly endorsed and all concerns about objective events will not be. More exaggerated subjective responses—such as that one would be perturbed for the whole week following a fearful encounter—might not be endorsed upon reflection. Some objectively dangerous outcomes might also be particularly interesting, especially those that combine subjective and objective elements. This is reflected in the idea that the needle phobic people might lose control and therefore be stabbed in the wrong place, which seemed to be somewhat differently affected by the bet manipulation than the other two objective events in Experiment 1. In clinical sessions, we have found that height phobic people are often concerned they might be so afraid that they lose control and fall or even jump from high places. The extremity of some subjective experiences might provoke concerns about objective threat that are caused by subjective responses, which could undermine a person’s ability to safely deal with a situation (e.g., extreme trembling when climbing a ladder). Further indirect effects on perception of objective threats might be produced through the simulation and embodiment of anticipated fearful feelings, which may invoke the actual fear state pre-emptively, in turn leading to heightened perceptions of threat due to emotional/*‘ex-consequentia’* reasoning^17,46^. These possibilities again highlight the potential importance of subjective expectations, as they may indirectly affect estimates of objective threat.

Our findings also help answer some open questions from previous behavioural experiments on this topic^24^. In those experiments, initial probability ratings were compared with monetary bets on the most-believed negative event really happening. High fear participants’ bets were lower than their corresponding probability ratings might imply, and although fearful participants gave much higher probability ratings than low fear participants, bets were similar between groups. One possibility would be that this probability vs. bet difference was an artifact of using a different rating type (0–100 probability vs. €0–€100), or merely reflected some kind of betting strategy. In the current experiments, participants who received the hypothetical bet instruction gave lower ratings than control participants using the exact same response format as one another. This bolsters the case that previously-observed differences between probability ratings and bets were not simply methodological artifacts.

Secondly, a full-fledged bet with actual money would not always be useful if pursued as a cognitive tool in therapy: it might be impractical and unethical to involve money in multiple therapy sessions, and the patient might find it difficult to incentivise themselves outside of sessions. The current findings suggest that invoking the mere idea of a bet on what would happen in reality can be a useful cognitive device for generating or remembering a more realistic perspective; a prospect we hope to test in phobic individuals. Thirdly, our previous experiments did not include subjective events, and we expected that fearful individuals might anticipate negative subjective experiences actually happening in a fear-provoking encounter. Our findings support the idea that fearful individuals are more convinced of subjectively negative things happening than they are of objectively threatening events occurring, and also that the subjective experience features prominently in their concerns about encounters with feared stimuli.

However, our findings do not suggest that all fearful individuals have total insight into the irrationality of their threat-related beliefs. In Experiment 1, among the people who reported a discrepancy between their initial ratings and their subsequent predictions of what would really happen, 91% said it was because they knew these events were unlikely in reality. Nevertheless, there was a sizable proportion of people who did not note such a discrepancy, despite similar responses to those who did. Many possibilities could explain this. People may simply have different thresholds for acknowledging such contradictions, or struggle to note the difference in probability, given that one rating used a 0–100 likelihood, whereas the other was a prediction of 0–50 phobic people experiencing the event. Alternatively, we have previously suggested that when participants respond with a probability rating, they might not really be thinking in terms of true probabilities, but rather just giving numbers that in some way represent to them the magnitude or impact of what they are thinking about^24^. Whatever the explanation, if such cognitive manipulations are used in therapy to help people generate insight into their negative expectations, some people might need to be more explicitly encouraged to put the two ratings side-by-side to recognise that they are inconsistent.

Alongside aforementioned limitations regarding how different beliefs or fear types might respond differently to the manipulations employed here, the sample we have collected is not a clinical sample. We intend to perform similar experiments with phobic patients to assess how they respond, and if these cognitive tools are found to be useful for them. In particular, it would be interesting to know whether an increased capacity for insight into the irrationality of certain threat expectations translates into changes in actual behaviour with feared stimuli. As a first step, however, responses from people regarding their self-reported fears can provide a testing ground for ideas that might be pursued in harder-to-reach clinical samples. There is little reason to believe that participants acquired through online sampling are any less reliable than those from typical subject pools or community samples^20–22^, especially given our stringent quality checks, the consistency of findings across the current experiments, and congruence with previous findings^24^. However, the sample is not representative of the United States population, or of any specific psychiatric population. For example, women were underrepresented in the needle experiments despite the higher prevalence of fear of needles among women than men^18^. This may be due to the even greater general prevalence, and sex difference, for fear of spiders and other animals^47^, causing women to neglect fear of needles upon seeing the fear of spiders option. The educational and ethnic backgrounds of the sample were also not representative, meaning that there may be sociocultural factors that are not captured in the present sample, and which may affect fear and fear-related beliefs.

Finally, we are limited in the breadth of claims that can be made with regards to the multitude of other beliefs that fearful individuals might hold, and which we did not ask about. It would be interesting to expand the range and extremity of subjective and objective events asked about, and to give participants the option of describing their own idiosyncratic concerns, to see if these can be similarly manipulated. Though not without its own limitations, directly investigating fear-related expectations in this way provides another lens for elucidating fear-related cognition and behaviour that is difficult to achieve using laboratory paradigms such as fear conditioning, in which new ‘fears’ are instilled in participants.

In summary, our findings highlight two interesting aspects of fear-related beliefs. Firstly, that a number of beliefs about objective threat–often held to be central in understanding why people are irrationally afraid^1^—are responsive to quite small cognitive manipulations. These cognitive manipulations might be pursued as means of helping fearful individuals to generate or recall more realistic or adaptive perspectives, which could impact fear directly or help fearful people to engage in exposure exercises. Secondly, beliefs about negative subjective experiences during fear-provoking encounters appear to be more strongly endorsed and feature prominently in fearful individuals’ concerns about what will happen in a fearful situation. Such direct approaches to understanding fear-related cognitions might help elucidate the complex nature of belief in fears and phobias.

## Supplementary Information

Supplementary Information.

## Data Availability

All code and data for analyses is available at: https://osf.io/x4j5n/?view_only=8a61ed7eb1704e0ca859d14a38c38d8e.
