# Supplementary material for: Expectations of objective threats and aversive feelings in specific fears
Source: Sci Rep. 2021 Oct 21;11:20778. doi: 10.1038/s41598-021-00317-3 (PMC8531133; doi:10.1038/s41598-021-00317-3)
Supplement: Supplementary file 1 — Supplementary Information. [file 41598_2021_317_MOESM1_ESM.pdf]

# Supplementary Materials for "Expectations of objective threats and aversive feelings in specific fears"

James W. B. Elsey, Merel Kindt

## Probability of Superiority

On top of the main paper in which we describe what Probability of Superiority (PSup) is, we here provide a couple of further illustrations to guide the reader. Recall that PSup is simply the probability that if we draw a random observation from one group or condition 'A', and compare it with another random observation from a comparison group or condition 'B', that A will be greater than B. In a within-subjects comparison, it is the proportion of subjects in whom the score in Condition A is greater than Condition B. Readers may be used to thinking of pairwise comparisons in terms of a metric such as Cohen's *d* (the difference between groups, normalized by the pooled standard deviation). When data is normally distributed and with equal variances, we can directly transform Cohen's *d* to a corresponding PSup value, as shown in Figure S1. We do this to help readers who are used to thinking in terms of Cohen's *d* to get a sense of the size of the effects indicated by our PSup values in the main paper. However, we would also encourage the reader to think directly in terms of the PSup probabilities – although there are some rules of thumb about *d* values being small, medium, or large, it is hard to understand the implications of *d* values if not thinking in terms of the likelihood of participants in one group scoring more highly than those of the other, which is exactly what PSup provides directly.

Often, we might also judge the size of an effect by assessing to what degree it is simply visible to our naked eye. For this reason, we included the raw data in the main text, with cumulative distributions to convey differences between groups. As people might instead be used to looking at density plots or histograms of data, below we include an example from Experiment 3 Spider analyses presented as a density plot (Figure S2). PSup values are

presented in each graph so the reader can get a direct sense of what different sizes mean from their own perspective of what looks like a meaningful result.

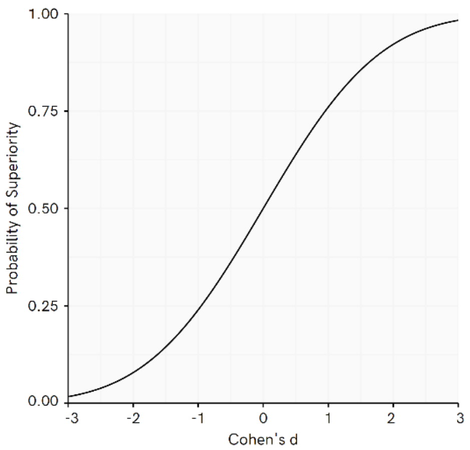


Figure S1. Probability of Superiority and corresponding Cohen's *d* values for normality distributed groups with equal variance.

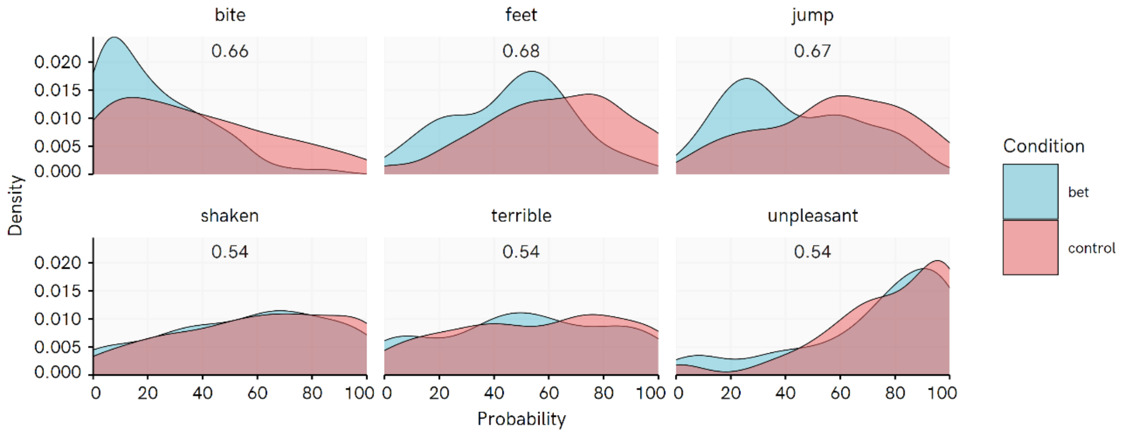


Figure S2. Density plots and probability of superiority for each event in Experiment 2, Tier 2. The upper distributions appear clearly distinguishable from one another and have correspondingly higher probability of superiority values than the distributions depicted in the lower panels.

**Exclusion criteria and MTurk settings**

Scoring for exclusion criteria was as follows:

- Making sense/being accurate when describing the task (0 or 1) [some scores were given .5 if they seemed to make sense but could not be guaranteed – in this way these participants could be included in Tier 1 analyses rather than completely excluded.

Task descriptions were replaced with a multiple choice format in Experiments 3 and 4 to remove this possible source of confusion]

- Correctly identifying the bet instruction (0 or 1) [for Experiments 3 and 4].
- Giving year of birth and age that correspond (0 or 1)
- Identifying consistently as from the US (0 or 1)
- Passing attention check questions about task features (0 - none, 1 - one right, 2 = 2 right)
- Completing all necessary tasks (0 or 1)

These were multiplied together to = 0 (total exclusion), 1 (not fully trustworthy but could be explored by others – we present analyses including Tier 1 participants in the supplement), 2 = passes all checks and included in analyses reported in main text). These exclusion criteria were included in our preregistration.

We also screened out participants from participating in the first instance if their IP addresses were identified as possibly belonging to IP farms or from other countries. Criteria were also set on MTurk for who the study was visible to, as shown in Table S1.

Table S1. MTurk recruitment criteria for each batch.

| Batch | Date | Approval | Previously completed tasks | Desired n per batch | Experiments |
|---|---|---|---|---|---|
| 1 | 28/09/2020 | >98 | 100 | 100 | 1, 2, 5 |
| 2 | 30/09/2020 | >98 | 100 | 100 | 1, 2, 5 |
| 3 | 01/10/2020 | >98 | 100 | 150 | 1, 2, 5 |
| 4 | 02/10/2020 | >98 | 100 | 150 | 1, 2, 5 |
| 5 | 26/10/2020 | >99 | 100 | 100 | 3, 4, 5 |
| 6 | 27/10/2020 | >99 | 100 | 100 | 3, 4, 5 |
| 7 | 28/10/2020 | >98 | 500 | 100 | 3, 4, 5 |
| 8 | 29/10/2020 | >98 | 500 | 100 | 3, 4, 5 |
| 9 | 02/11/2020 | >98 | 500 | 100 | 3, 4, 5 |
| 10 | 16/11/2020 | >98 | 500 | 100 | 4, 5 |
| 11 | 17/11/2020 | >98 | 500 | 100 | 4, 5 |
| 12 | 16/02/2021 | >98 | 500 | 100 | 4, 5 |
| 13 | 17/02/2021 | >98 | 500 | 100 | 4, 5 |
| 14 | 18/02/2021 | >98 | 500 | 100 | 4, 5 |

**Priors**

**Experiment 1**

probability ~ 0 + fear level * bet vs. belief condition * event + (1 | ppn)

Each cell of the interaction given prior (set on logit scale):

normal(m = 0 , sd = 1.4)

Phi given prior:

student_t(nu = 5, m = 0 , sd = 5)

**Experiments 2-5**

probability ~ 0 + bet vs. control condition * event + questionnaire z score + (1 | ppn)

Each cell of the interaction given prior (set on logit scale):

normal(m = 0 , sd = 1.4)

Questionnaire z score given prior (set on logit scale):

student_t(nu = 5, m = 0 , sd = 1.25)

Phi given prior:

student_t(nu = 5, m = 0 , sd = 5)

**Sensitivity Analyses**

Analyses were also conducted on data with the inclusion of Tier 1 participants, and an analysis of Experiment 1 with non-overlapping IPS scores. Plots for all these analyses are presented below. As with the main analyses, code and data files are available for each analysis at https://osf.io/x4j5n/?view_only=8a61ed7eb1704e0ca859d14a38c38d8e

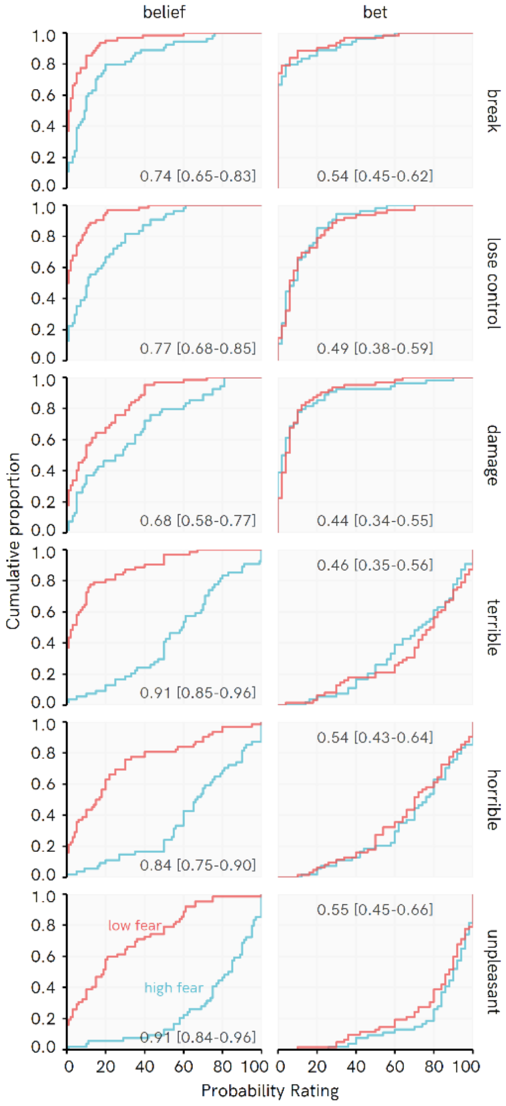


Figure S3. Cumulative distribution for Experiment 1, exclusive fear groups, with raw probability of superiority value and bootstrapped 95% bias corrected accelerated confidence intervals.

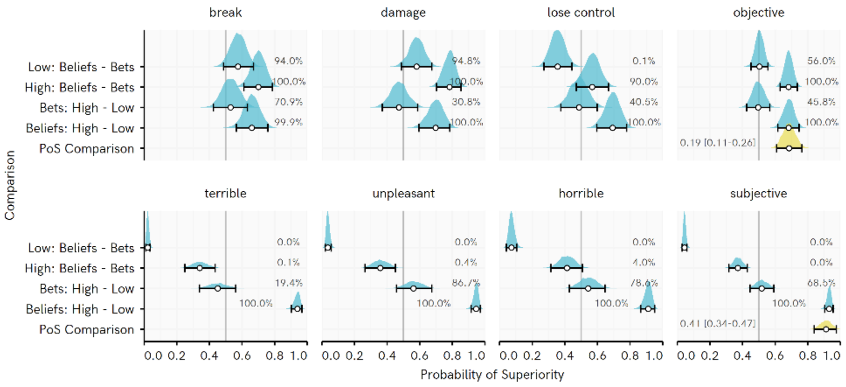


Figure S4. Posterior distribution of regression-based probability of superiority (PSup) estimates for Experiment 1, exclusive fear groups. Percentages indicate the proportion of the posterior PSup estimate >.5. Points represent posterior median, whiskers represent 95% HDI, ridge height reflects density of estimate. PoS Comparison = difference in PSup estimates for Beliefs: High - Low *vs.* Bets: High - Low, with values above 0 indicating a greater difference between High and Low fear participants in Beliefs than Bets, with posterior median and [95% highest density interval]. Note that position of this density ridge on the X axis has been shifted by .5 so that the line at .5 represents 0 for this posterior distribution.

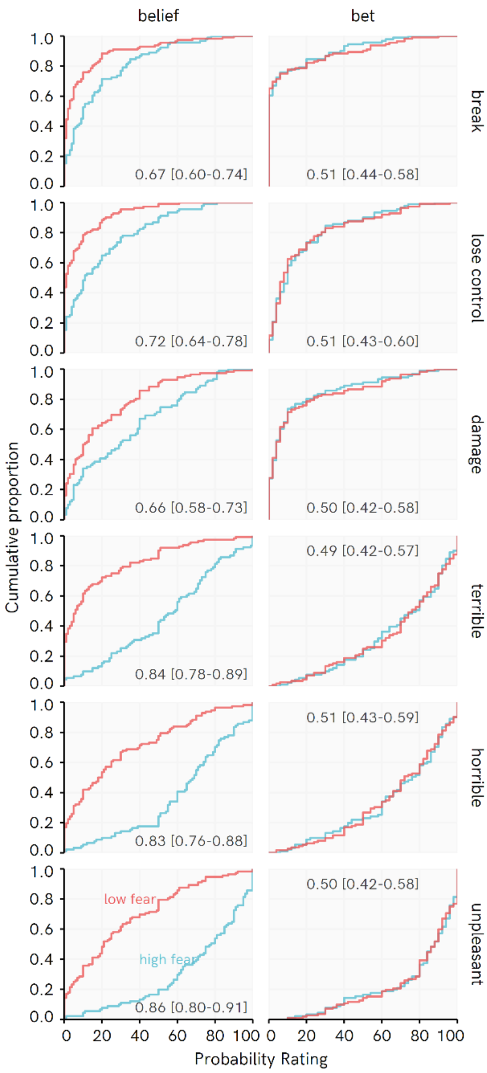


Figure S5. Cumulative distribution for Experiment 1, Tier 1 & 2 participants, with raw probability of superiority value and bootstrapped 95% bias corrected accelerated confidence intervals.

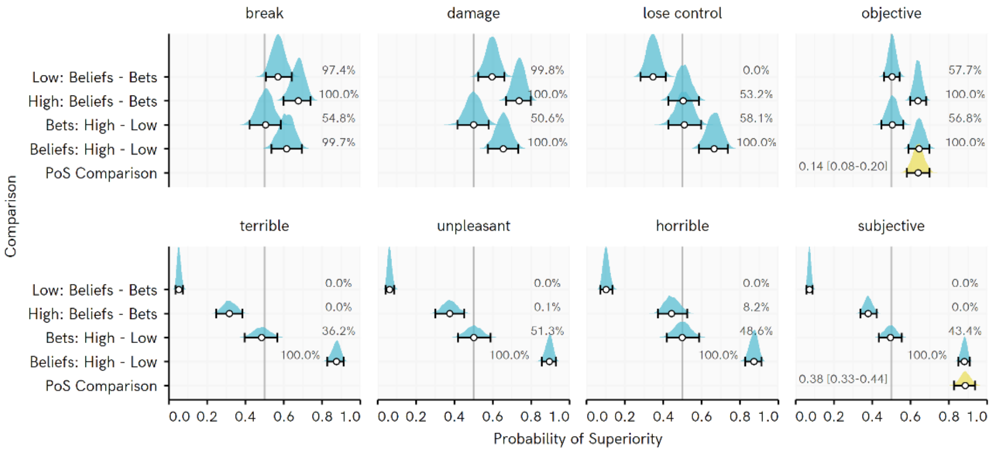


Figure S6. Posterior distribution of regression-based probability of superiority (PSup) estimates for Experiment 1Tier 1 & 2 participants. Percentages indicate the proportion of the posterior PSup estimate >.5. Points represent posterior median, whiskers represent 95% HDI, ridge height reflects density of estimate. PoS Comparison = difference in PSup estimates for Beliefs: High - Low *vs.* Bets: High - Low, with values above 0 indicating a greater difference between High and Low fear participants in Beliefs than Bets, with posterior median and [95% highest density interval]. Note that position of this density ridge on the X axis has been shifted by .5 so that the line at .5 represents 0 for this posterior distribution.

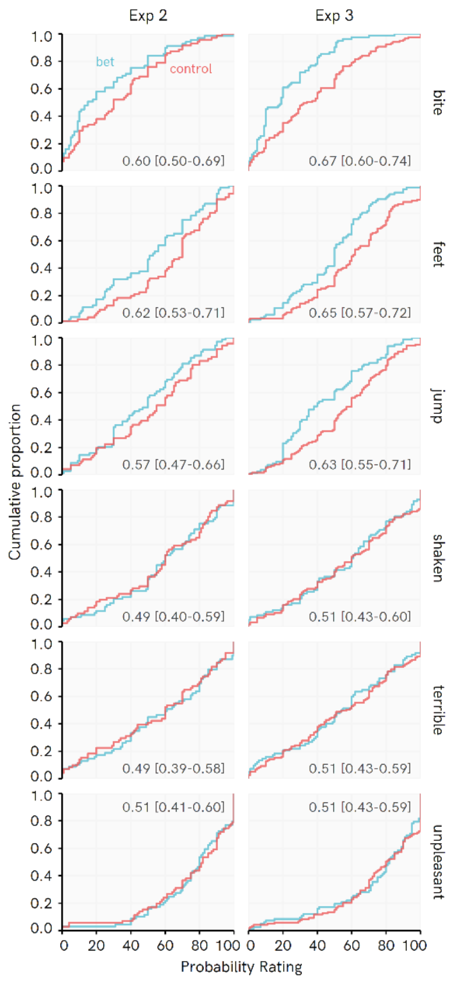


Figure S7. Cumulative distributions for Experiments 2 and 3, Tier 1 & 2 participants, with raw probability of superiority value and bootstrapped 95% bias corrected accelerated confidence intervals.

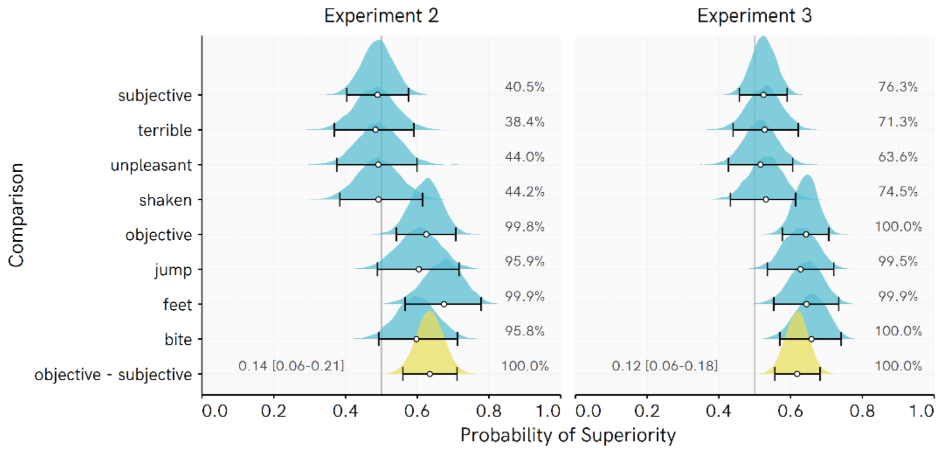


Figure S8. Posterior distribution of regression-based probability of superiority (PSup) estimates for Experiments 2 and 3, Tier 1 and 2 participants. Each PSup value represents an estimate of the probability that a randomly picked Control score is higher than a randomly picked Bet score. Percentages indicate the proportion of the posterior PSup estimate >. Points represent posterior median, whiskers represent 95% HDI, ridge height reflects density of estimate. Objective - Subjective = difference in PSup estimates for average of Objective beliefs vs. the average of Subjective beliefs, with values above 0 indicating a greater difference between Control and Bet participants in Objective than Subjective beliefs, with posterior median and [95% highest density interval]. Note that position of this density ridge on the X axis has been shifted by .5 so that the line at .5 represents 0 (no difference in PSup estimates) for this posterior distribution.

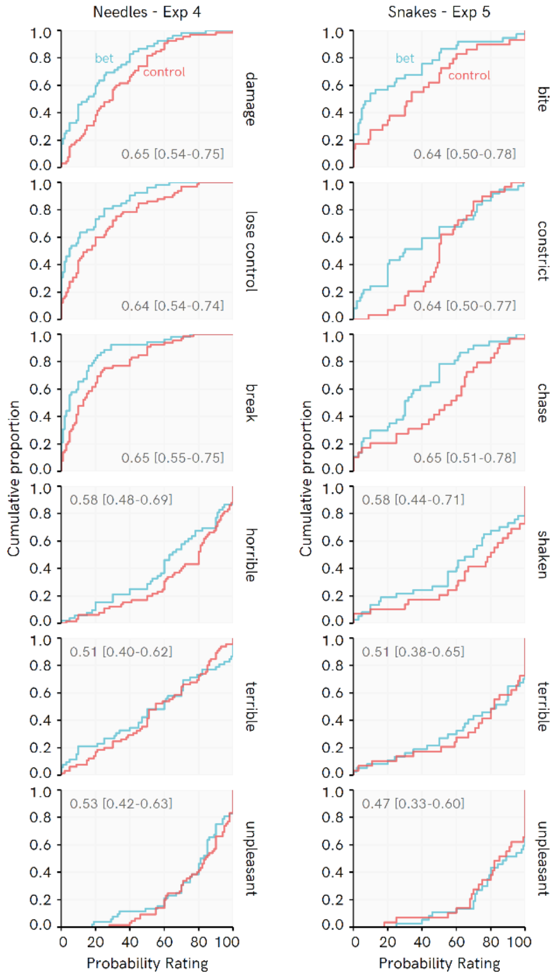


Figure S9. Cumulative distributions for Experiments 4 and 5, Tier 1 & 2 participants, with raw probability of superiority value and bootstrapped 95% bias corrected accelerated confidence intervals.

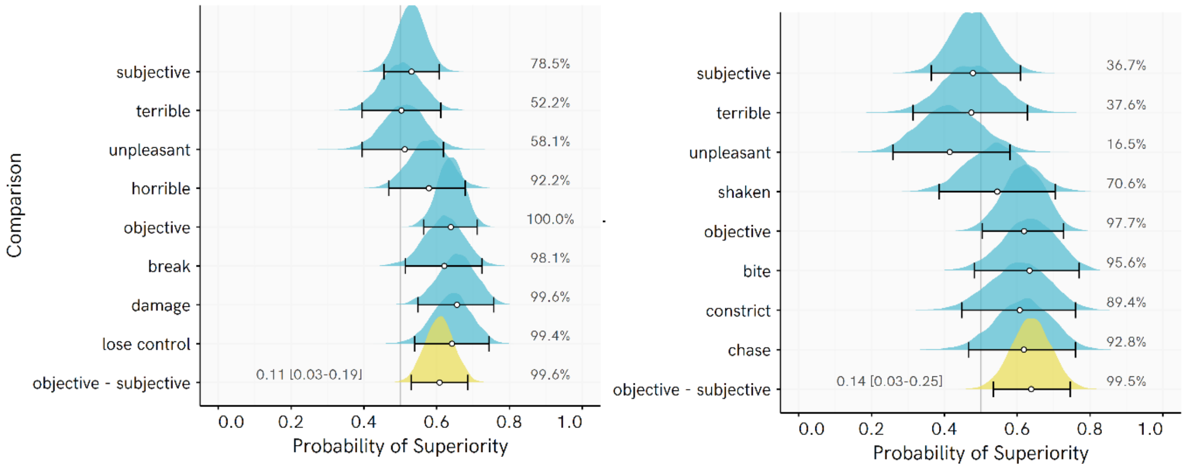


Figure S10. Posterior distribution of regression-based probability of superiority (PSup) estimates for Experiments 4 (left) and 5 (right), Tier 1 and 2 participants. Each PSup value represents an estimate of the probability that a randomly picked Control score is higher than a randomly picked Bet score. Percentages indicate the proportion of the posterior PSup estimate >. Points represent posterior median, whiskers represent 95% HDI, ridge height reflects density of estimate. Objective - Subjective = difference in PSup estimates for average of Objective beliefs vs. the average of Subjective beliefs, with values above 0 indicating a greater difference between Control and Bet participants in Objective than Subjective beliefs, with posterior median and [95% highest density interval]. Note that position of this density ridge on the X axis has been shifted by .5 so that the line at .5 represents 0 (no difference in PSup estimates) for this posterior distribution.

## Interim Analysis

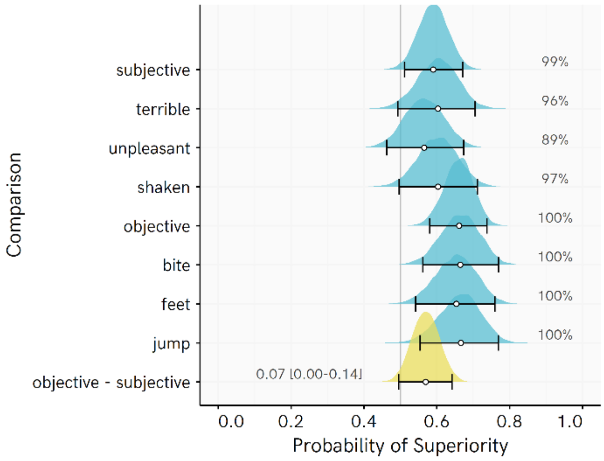


Figure S11. Posterior distribution of regression-based probability of superiority (PSup) estimates for Experiment 3 at the interim analysis. Each PSup value represents an estimate of the probability that a randomly picked Control score is higher than a randomly picked Bet score. Percentages indicate the proportion of the posterior PSup estimate >. Points represent posterior median, whiskers represent 95% HDI, ridge height reflects density of estimate. Objective - Subjective = difference in PSup estimates for average of Objective beliefs vs. the average of Subjective beliefs, with values above 0 indicating a greater difference between Control and Bet participants in Objective than Subjective beliefs, with posterior median and [95% highest density interval]. Note that position of this density ridge on the X axis has been shifted by .5 so that the line at .5 represents 0 (no difference in PSup estimates) for this posterior distribution.

An interim analysis was performed for Experiment 3, at which point the posterior distribution for the difference in Probability of Superiority for Objective vs. Subjective beliefs just tipped over 0. Data collection therefore continued for 2 additional batches for Experiment 3. No other interim analyses were performed because sample sizes had not reached the desired number for other experiments.

## Additional information on regression models

All regression models used in analyses are available as RData files available at https://osf.io/x4j5n/?view_only=8a61ed7eb1704e0ca859d14a38c38d8e. In these files, chain diagnostics such as Rhat show that all chains converged, and there were good effective sample sizes for all parameters. Files on the OSF also include CSV/Excel files that provide simple summary statistics from the regression models such as the means for each condition/group/event, on both the logit and direct probability scale. We recommend using the

raw data plots in the main text and corresponding median and MAD values for a more descriptive understanding of the data.

## Stimuli

### Task description for Experiment 1 (Needles)

On the next page, you will find a **description of a situation involving an injection/blood draw**. You will then be given a list of several **events that people might be afraid of happening if they were in the situation**. Your task will be to **rate how much you think those events might happen to you if you were in this situation -** i.e., the probability of those events, from 0 ("this will certainly not happen") to 100 ("this will certainly happen"). 50 would mean you think it is equally likely that the event would happen as that it would not happen. Please note that you can use the digital slider to select ANY number from 0-100 to indicate your response.

Please answer honestly - we really want to know what you think.

**Imagine that you have been required to receive a blood draw or injection (whichever you are more afraid of).** A trained healthcare professional will administer the blood draw/injection by inserting a needle into a vein in your arm.

Several things that people might be afraid of happening when having a blood draw/injection are listed on the next page.

We would like to know to what extent you think these things might happen if you were required to receive a blood draw/injection.

You can use the slider to choose any number from 0 ("this will certainly not happen") to 100 ("this will certainly happen"). 50 would mean you think it is equally likely that the event would happen as that it would not happen. Please answer honestly, we really want to know what you think.

### Events for Experiment 1 (Needles)

- I will be so scared that it just feels absolutely terrible
- For some reason, the needle will miss the target vein and my arm will be damaged or I will bleed a lot
- I will lose control of my body, causing the needle to stab me in the wrong place
- Something will go wrong and the needle will break off or otherwise get stuck in my arm
- I will have unpleasant feelings in my body, such as my heart racing or stomach churning
- I will find the sense of the needle going in my arm to be completely horrible

### Bet task description for needle participants

**In the following task, it is possible for you to win 25 dollars as a bonus to your standard payment.** The winner will be selected based upon who has **the most accurate responses**. Therefore, please read the following instructions carefully.

At the University of Amsterdam, we have been running treatments for people with fear of needles/injections. A key part of the treatment is a **brief but fear-provoking exposure to needles**.

**We recently conducted a study with 50 patients who had a phobia (extreme fear) of needles/injections**.

Each patient was required to undergo either **an injection or a blood draw - whichever they feared the most. A trained nurse gave the injection/blood draw. The psychotherapist waited in a separate room.**

In order to win the 25 dollars, we would like you to **estimate how many of the 50 patients experienced the events listed on the following page**. For each item, **you can choose any number from 0 to 50, to indicate the number of patients you think that event happened to**. **Whether you are right or wrong is determined by what actually happened in reality when we did the experiment**.

**The people with the most accurate estimates can win the 25 dollars:** Six people with the most accurate responses will be selected to win this additional money. If several people score equally well, the winner will be randomly determined from them.

**Bet events for Experiment 1 (Needles)**

- When receiving the blood draw/injection, *how many needle phobic patients do you think found the experience to be terrible (patients reported their feelings shortly afterwards)?* You can choose any number from 0 to 50.
- When receiving the blood draw/injection, *for what number of patients do you think the needle missed the intended vein and damaged their arm or made them bleed a lot?* You can choose any number from 0 to 50.
- When receiving the blood draw/injection, *how many patients do you think lost control and moved so much that the needle stabbed them in the wrong place?* You can choose any number from 0 to 50.
- When receiving the blood draw/injection, *for how many needle phobic patients do you think the needle broke or otherwise got stuck in their arm?* You can choose any number from 0 to 50.
- When receiving the blood draw/injection, *how many needle phobic patients do you think reported feeling unpleasant bodily sensations, such as their heart pumping fast or feeling sick (patients were asked about their sensations afterwards)?* You can choose any number from 0 to 50.
- When receiving the blood draw/injection, *how many needle phobic patients do you think found the sense of the needle going into their arm to be totally horrible (patients were asked about this experience shortly afterwards)?* You can choose any number from 0 to 50.

**Feelings vs. danger question for Experiment 1 (Needles)**

If you think about receiving a blood draw/injection, what do you find worse – if you had to pick one or the other?:

- The danger posed by the needle/injection
- How the process would make me feel

**Additional questions for Experiment 1 (Needles)**

We have noticed that some people with fear of needles/injections rate events about how the needle might harm them or pose a real threat to be quite likely for themselves. When asked to predict how many patients this might happen to in real life, their estimate seems much lower. For example, they might give a high rating that the needle will miss their vein and damage their arm. When asked to predict how many patients this will happen to, they might say none or very few.

Do you think you had a similar mismatch between what you thought might happen to yourself versus other people? If yes, which statement best describes your thoughts on why this is?

- No
- Possibly/Yes: Because bad things are really more likely to happen to me than to other phobic people
- Possibly/Yes: Because when I think about these things really happening, I know they are unlikely in reality

**Task description for Experiments 2 and 3 (Spiders)**

On the next page, you will find a **description of a situation involving a spider**. You will then be given a list of several **events that people might be afraid of happening if they were in the situation**. Your task will be to **rate how much you think those events might happen to you if you were in this situation -** i.e., the probability of those events, from 0 ("this will certainly not happen") to 100 ("this will certainly happen"). 50 would mean you think it is equally likely that the event would happen as that it would not happen. Please note that you can use the digital slider to select ANY number from 0-100 to indicate your response.

Please answer honestly **-** we really want to know what you think.

**[Additional instruction given to Bet participants:**

**We would like you to think about your answers in a particular way:** Imagine that for each event that might happen, you had to place a bet on what would actually happen in reality in the situation. You could then win money by correctly predicting what will really happen. Before answering what you think will happen, please think about whether you would bet money on the event actually happening in reality, then give your answer.]

**Imagine that you have been asked by a researcher to take part in an 'exposure task' with a real spider.** In this task, you will **enter a room on your own, in your bare feet** (no shoes or socks). In the middle of the room, **there is a house spider on the floor** (6-7cm from its front to back legs). This house spider is a **'wild' spider that has just been caught by the researcher, but it is not venomous.**

You are given a small paint brush with which to touch the spider. **Your task will be to approach within 30 centimeters of the spider and touch it with the brush, to see what happens.** You must stay standing where you are when you touch the spider.

Several things that people might be afraid of happening when they do the exposure task are presented on the next page.

We would like to know to what extent you think these things might happen if you had to perform the exposure task.

You can use the slider to choose any number from 0 ("this will certainly not happen") to 100 ("this will certainly happen"). 50 would mean you think it is equally likely that the event would happen as that it would not happen. Please answer honestly, we really want to know what you think.

[Reminder given to Bet participants:

REMEMBER – before answering each question in this section, **think:** *Would I bet money on the event actually happening in reality?* Then give your answer.]

**Events for Experiments 2 and 3 (Spiders)**

- The spider will climb up the brush and onto my hand
- The spider will run onto my bare feet
- The spider will bite me

- I will be so scared that it just feels absolutely terrible
- After the exposure task I will be shaken and upset
- I will have unpleasant feelings in my body, such as my heart racing or stomach churning

**Feelings vs. danger question for Experiments 2 and 3 (Spiders)**

If you think about being exposed to a non-venomous spider, what do you find worse – if you had to pick one or the other?:

- The danger posed by the spider
- The way the spider makes me feel

**Additional questions for Experiments 2 and 3 (Spiders)**

If you think about the task, do you believe that you could do it? (from ‘Definitely could NOT do it’, to ‘Definitely COULD do it’)

How afraid would you be if you had to perform the task? (from ‘No fear at all’, to ‘Worst fear imaginable’)

**Task description for Experiment 4 (Needles)**

On the next page, you will find a **description of a situation involving an injection/blood draw**. You will then be given a list of several **events that people might be afraid of happening if they were in the situation**. Your task will be to **rate how much you think those events might happen to you if you were in this situation -** i.e., the probability of those events, from 0 ("this will certainly not happen") to 100 ("this will certainly happen"). 50 would mean you think it is equally likely that the event would happen as that it would not happen. Please note that you can use the digital slider to select ANY number from 0-100 to indicate your response.

Please answer honestly - we really want to know what you think.

**[Additional instructions for Bet participants:**

**We would like you to think about your answers in a particular way:** Imagine that for each event that might happen, you had to place a bet on what would actually happen in reality in the situation. You could then win money by correctly predicting what will really happen. Before answering what you think will happen, please think about whether you would bet money on the event actually happening in reality, then give your answer.]

**Imagine that you have been asked by a researcher to take part in an ‘exposure task’ involving a real blood draw/injection (whichever you are more afraid of).** In this task, **the researcher will wait in another room. You will enter a testing room with a trained healthcare professional. The healthcare professional will administer the blood draw/injection by inserting a needle into a vein in your forearm.**

Several things that people might be afraid of happening when they do the exposure task are presented on the next page.

We would like to know to what extent you think these things might happen if you had to perform the exposure task.

You can use the slider to choose any number from 0 ("this will certainly not happen") to 100 ("this will certainly happen"). 50 would mean you think it is equally likely that the event would happen as that it would not happen. Please answer honestly, we really want to know what you think.

[Reminder given to Bet participants:

REMEMBER – before answering each question in this section, **think:** *Would I bet money on the event actually happening in reality?* Then give your answer.]

**Events for Experiment 4 (Needles)**

- I will be so scared that it just feels absolutely terrible
- For some reason, the needle will miss the target vein and my arm will be damaged or I will bleed a lot
- I will lose control of my body, causing the needle to stab me in the wrong place
- Something will go wrong and the needle will break off or otherwise get stuck in my arm
- I will have unpleasant feelings in my body, such as my heart racing or stomach churning
- I will find the sense of the needle going in my arm to be completely horrible

**Feelings vs. Danger Question for Experiment 4 (Needles)**

If you think about receiving a blood draw/injection, what do you find worse – if you had to pick one or the other?:

- The danger posed by the needle/injection
- How the process would make me feel

**Additional questions for Experiment 4 (Needles)**

If you think about the task, do you believe that you could do it? (from 'Definitely could NOT do it', to 'Definitely COULD do it')

How afraid would you be if you had to perform the task? (from 'No fear at all', to 'Worst fear imaginable')

**Task description for Experiment 5 (Snakes)**

On the next page, you will find a **description of a situation involving a snake**. You will then be given a list of several **events that people might be afraid of happening if they were in the situation**. Your task will be to **rate how much you think those events might happen to you if you were in this situation -** i.e., the probability of those events, from 0 ("this will certainly not happen") to 100 ("this will certainly happen"). 50 would mean you think it is equally likely that the event would happen as that it would not happen. Please note that you can use the digital slider to select ANY number from 0-100 to indicate your response.

Please answer honestly - we really want to know what you think.

[Additional instructions given to Bet participants:

**We would like you to think about your answers in a particular way:** Imagine that for each event that might happen, you had to place a bet on what would actually happen in reality in the situation. You could then win money by correctly predicting what will really happen. Before answering what you think will happen, please think about whether you would bet money on the event actually happening in reality, then give your answer.]

**Imagine that you have been asked by a researcher to take part in an 'exposure task' with a real snake.** In this task, you will enter a room. **In the middle of the room, there is a boa constrictor snake.** This snake is greenish-brown and black in color, and is **5 feet long (about 1.5 meters). The**

**snake is used in demonstrations at a local zoo to allow the public to interact with real snakes.** Boa constrictor snakes are **not venomous, but catch their prey such as rodents by 'constricting' them**/wrapping around them. **The zoo instructor will be in the room but will stand away from both you and the snake** during the exposure task.

Your task will be to **approach within 18 inches (45cm) of the snake's head, and stay standing there for 1 minute, to see what happens.** If the snake moves, you can also move, but **you must keep within 18 inches of the head of the snake for one minute.**

Several things that people might be afraid of happening when they do the exposure task are presented on the next page.

We would like to know to what extent you think these things might happen if you had to perform the exposure task.

You can use the slider to choose any number from 0 ("this will certainly not happen") to 100 ("this will certainly happen"). 50 would mean you think it is equally likely that the event would happen as that it would not happen. Please answer honestly, we really want to know what you think.

[Reminder given to Bet participants:

REMEMBER – before answering each question in this section, **think:** *Would I bet money on the event actually happening in reality?* Then give your answer.]

**Events for Experiment 5 (Snakes)**

- The snake will come after me and try to catch me
- The snake will start wrapping itself around my leg
- The snake will bite me
- I will be so scared that it just feels absolutely terrible
- After the exposure task I will be shaken and upset
- I will have unpleasant feelings in my body, such as my heart racing or stomach churning

**Feelings vs. Danger Question for Experiment 5 (Snakes)**

If you think about being exposed to a non-venomous snake, what do you find worse – if you had to pick one or the other?:

The danger posed by the snake – The way the snake makes me feel

**Additional questions for Experiment 5 (Snakes)**

If you think about the task, do you believe that you could do it? (from 'Definitely could NOT do it', to 'Definitely COULD do it')

How afraid would you be if you had to perform the task? (from 'No fear at all', to 'Worst fear imaginable')
